# Targeted silencing of CLYBL with platelet-mimetic siRNA nanoparticles drives itaconate–mediated macrophage reprogramming and protects against sepsis-triggered lung cell death

**DOI:** 10.1038/s41420-026-03119-6

**Published:** 2026-05-30

**Authors:** ZuoJun Huang, Jialin Zhong, Li Zhang, Xianggui Huang, Shanshan Liang, Youfeng Zhu, Rui Zhang, Hongzhi He, Chengcheng Xu, Wang Chen, Jing Wang, Xiaolong Wu, Yumin Liang, Jian Zou, Shuyao Zhang

**Affiliations:** 1 Department of Pharmacy, Guangzhou Red Cross Hospital of Jinan University, Guangzhou, PR China; 2 Department of Pediatrics, Guangzhou Red Cross Hospital of Jinan University, Guangzhou, PR China; 3 Department of Intensive Care Unit, Guangzhou Red Cross Hospital of Jinan University, Guangzhou, PR China; 4 Department of Emergency Medicine, Guangzhou Red Cross Hospital of Jinan University, Guangzhou, PR China

**Keywords:** Molecular biology, Cell biology

## Abstract

Excessive inflammation and metabolic dysregulation fuel alveolar cell death in sepsis-induced lung injury, yet effective molecular interventions are lacking. We identify citrate lyase beta-like (CLYBL) as a previously unrecognized metabolic driver of macrophage-mediated tissue damage. In a murine cecal ligation and puncture model, CLYBL was strongly upregulated in lung tissue and peritoneal macrophages. To therapeutically target this pathway, we engineered platelet-derived extracellular vesicle–coated poly(lactic-co-glycolic acid) nanoparticles (PEVs@PLGA) encapsulating CLYBL-specific small interfering RNA. This platelet-mimetic system enabled efficient, biocompatible delivery of siRNA and robust CLYBL knockdown both in vitro and in vivo. CLYBL silencing triggered accumulation of the anti-inflammatory metabolite itaconate, limited M1 macrophage polarization, and preserved alveolar epithelial integrity, thereby reducing cell death and improving pulmonary repair. Transcriptomic analysis revealed broad immunometabolic remodeling consistent with enhanced resolution of inflammation. Biosafety evaluation confirmed negligible systemic toxicity. These findings uncover CLYBL as a critical metabolic checkpoint linking macrophage activation to alveolar cell death and highlight platelet-mimetic siRNA nanoparticles as a potent therapeutic strategy. Our work provides a mechanistic and translational framework for targeting macrophage immunometabolism to prevent fatal organ damage during sepsis.

**Figure:**
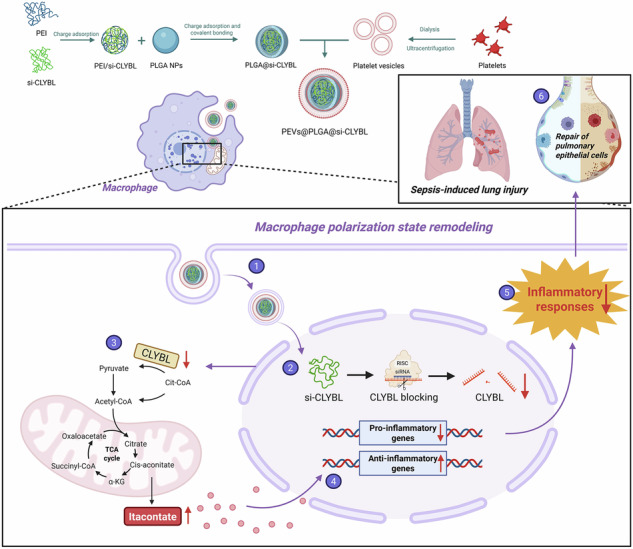
PEVs@PLGA@si-CLYBL promote itaconate accumulation, induce immune cell functional remodeling, and facilitate lung epithelial repair, offering a novel therapeutic approach for sepsis-induced lung injury (Created with BioRender.com).

PEVs@PLGA@si-CLYBL promote itaconate accumulation, induce immune cell functional remodeling, and facilitate lung epithelial repair, offering a novel therapeutic approach for sepsis-induced lung injury (Created with BioRender.com).

## Introduction

Sepsis, a systemic inflammatory response syndrome triggered by infection, is one of the leading causes of death in intensive care units (ICUs) [1–3]. Lung injury is the earliest and most common complication of sepsis and can rapidly progress to acute respiratory distress syndrome (ARDS), severely affecting patient outcomes [4, 5]. Currently, clinical management lacks effective targeted interventions with well-defined mechanisms, relying primarily on anti-infective therapies and organ support [6, 7]. These complex processes, particularly under the dynamic shift between cytokine storms and immunosuppression, limit the efficacy of conventional anti-inflammatory treatments [8–10]. Therefore, developing novel strategies targeting the intersection of immune and metabolic regulation holds significant clinical and translational value for improving sepsis-induced lung injury.

Macrophages play a pivotal role in sepsis-induced lung injury, serving as key immune cells that determine the intensity and resolution of the inflammatory response. They can polarize into either pro-inflammatory M1 or anti-inflammatory/tissue-repairing M2 phenotypes in response to microenvironmental cues [11–13]. In the early stage of sepsis, M1 macrophages are predominantly activated, leading to excessive cytokine release and exacerbation of lung tissue damage [11, 14]. Immunometabolic regulation is a critical driver of macrophage polarization, with metabolic intermediates not only providing energy but also functioning as signaling molecules to modulate gene expression and cellular functions [12, 15, 16]. Itaconate, a key immunometabolite, has been shown to suppress M1 polarization and alleviate inflammatory damage [17–19]. Enhancing intracellular levels of itaconate and modulating its metabolic pathway may offer a novel therapeutic avenue for controlling the inflammatory response and promoting lung tissue repair during sepsis.

Citrate lyase beta-like (CLYBL) is a widely expressed mitochondrial enzyme involved in the C5-dicarboxylic acid metabolic pathway [20, 21]. Previous studies suggest that CLYBL may regulate the citrate metabolic pathway and thereby influence the synthesis and accumulation of itaconate [22, 23]. It has been reported that CLYBL is significantly enriched and upregulated in macrophages from patients with pneumonia, where it depletes the immunoregulatory metabolite itaconate, thus impairing its anti-inflammatory function [23].

Small interfering RNA (siRNA) is a powerful tool for precise gene silencing, holding broad potential for disease treatment. However, its poor stability and low in vivo targeting efficiency necessitate the use of efficient delivery systems [24–26]. Due to their excellent encapsulation capacity and biodegradability, PLGA nanoparticles have been widely employed for the delivery of nucleic acids [27, 28]. In this study, we further developed a hybrid membrane delivery platform by coating PLGA nanoparticles with platelet-derived extracellular vesicles (PEVs), resulting in the construction of PEV-coated PLGA nanoparticles loaded with siRNA targeting CLYBL (PEVs@PLGA@si-CLYBL). Platelet membranes possess inherent targeting ability to inflammatory sites and immune evasion properties, which significantly enhance the efficiency and sustained release of siRNA in inflamed tissues. This system combines the structural stability of synthetic materials with the biological specificity of natural membranes, offering a viable platform for efficient and targeted siRNA delivery in vivo, with favorable biocompatibility and translational potential.

Based on the above theoretical foundation, this study aims to develop a PEVs@PLGA@si-CLYBL delivery system and to systematically explore its therapeutic potential in sepsis-induced lung injury. By establishing a cecal ligation and puncture (CLP) animal model, we aim to characterize the expression profile of CLYBL in lung tissues and macrophages, and achieve targeted silencing of CLYBL via the PEVs@PLGA@si-CLYBL delivery system. The study further investigates its regulatory effects on itaconate accumulation, macrophage polarization, and alveolar epithelial repair. In addition, transcriptomic analysis is employed to elucidate the underlying molecular mechanisms. This work is the first to propose CLYBL as a novel target for immunometabolic regulation and to establish an efficient and targeted nucleic acid delivery platform. It not only advances the mechanistic understanding of sepsis-induced lung injury but also provides a new technological strategy and theoretical basis for the application of nucleic acid therapeutics in immune-related diseases, with significant scientific and translational value.

## Results

### CLYBL is upregulated in macrophages during sepsis-induced lung injury

**Fig. 1:**
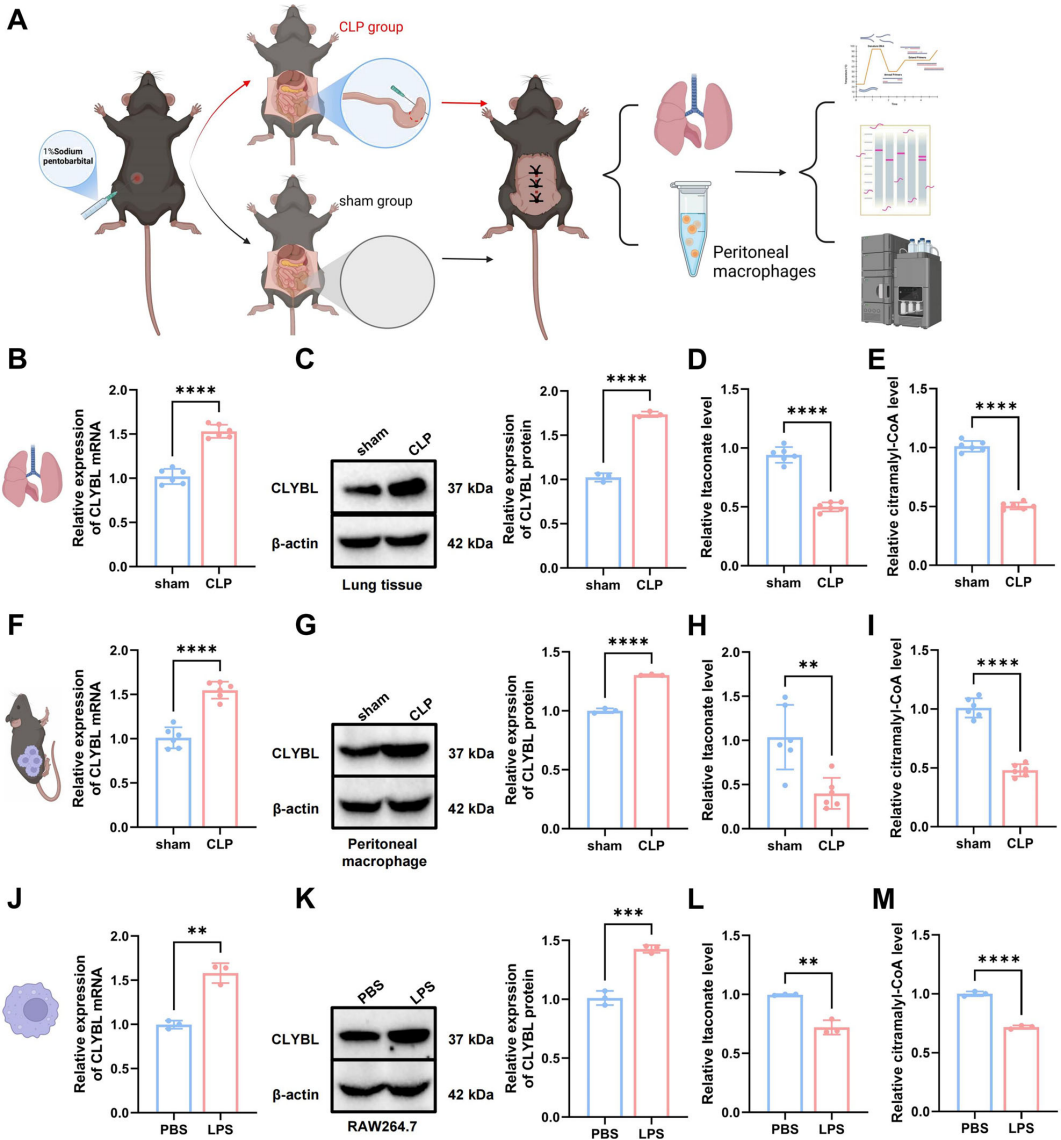
Altered expression of CLYBL in sepsis-induced lung injury and its association with itaconate metabolism. **A** Schematic diagram illustrating the establishment of the mouse model of sepsis-induced lung injury (Created with BioRender.com); **B** RT-qPCR analysis of CLYBL mRNA expression in lung tissues of CLP and sham mice; **C** Western blot analysis of CLYBL protein levels in lung tissues from CLP and sham groups; **D** High-resolution LC-MS analysis of itaconate levels in lung tissues from CLP and sham mice; **E** LC-MS quantification of citramalyl-CoA levels in lung tissues from CLP and sham mice; **F** RT-qPCR detection of CLYBL expression in peritoneal macrophages from CLP and sham mice; **G** Western blot analysis of CLYBL protein expression in peritoneal macrophages; **H** LC-MS measurement of itaconate levels in peritoneal macrophages from CLP and sham mice; **I** LC-MS analysis of citramalyl-CoA levels in peritoneal macrophages from CLP and sham mice; **J** RT-qPCR analysis of CLYBL expression in LPS- and PBS-treated RAW264.7 cells; **K** Western blot analysis of CLYBL protein levels in LPS- and PBS-treated RAW264.7 cells; **L** LC-MS analysis of itaconate levels in RAW264.7 cells following LPS or PBS treatment; **M** LC-MS analysis of citramalyl-CoA levels in RAW264.7 cells following LPS or PBS treatment. All cell experiments were performed in triplicate, and eight mice were included per group. *p < 0.05, **p < 0.01, ***p < 0.001, ****p < 0.0001.

We first established a mouse model of sepsis-induced lung injury (Fig. 1A) and examined the expression dynamics of CLYBL under these conditions. Experimental groups included a sham group (undergoing laparotomy without cecal ligation) and a CLP group (CLP to induce sepsis-induced lung injury). Following the successful establishment of the model, lung tissues were comprehensively analyzed for pulmonary edema and histopathological changes. Pulmonary edema assessment revealed a significantly increased lung W/D weight ratio in the CLP group compared to the sham group, indicating notable edema induced by sepsis (Fig. S1A). Histological analysis further confirmed severe inflammatory infiltration and tissue damage in the CLP group. H&E staining revealed disrupted lung architecture (Fig. S1B), while PAS staining showed epithelial cell shedding (Fig. S1C). Additionally, Evans blue dye extravasation demonstrated markedly increased vascular permeability in the CLP group (Fig. S1D), indicating enhanced vascular leakage due to sepsis. IHC analysis showed a significant reduction in Spc-1 staining intensity in the CLP group compared to the sham group (Fig. S1E), further supporting the presence of lung injury.

We next assessed CLYBL expression under septic conditions. Both RT-qPCR and Western blot analyses revealed a significant upregulation of CLYBL in the lung tissues of CLP mice, in contrast to the sham group (Fig. 1B, C). To further investigate the potential immunomodulatory role of CLYBL, we quantified the levels of itaconate and citramalyl-CoA via high-resolution LC-MS. The results demonstrated a substantial reduction in itaconate and citramalyl-CoA levels in the CLP group compared to the sham group, suggesting that elevated CLYBL expression may restrict the anti-inflammatory effects of itaconate by suppressing its metabolic pathway (Fig. 1D, E). Collectively, these findings suggest that the upregulation of CLYBL is closely linked to sepsis-induced lung injury and immune dysregulation and may play a crucial role in the pathogenesis of sepsis-related pulmonary damage.

In the CLP model, macrophages play a critical role in orchestrating the immune response. To investigate the functional relevance of CLYBL in macrophages during sepsis-induced lung injury, we first isolated peritoneal macrophages from both sham and CLP mice. RT-qPCR and Western blot analyses revealed a significant upregulation of CLYBL in macrophages from CLP mice compared to the sham group (Fig. 1F, G). Furthermore, high-resolution LC-MS analysis showed a marked reduction in itaconate and citramalyl-CoA levels in macrophages from the CLP group, suggesting that increased CLYBL expression may be closely associated with itaconate depletion (Fig. 1H, I).

To further confirm the role of CLYBL in macrophage function, we stimulated RAW264.7 murine macrophage cells with LPS and assessed the expression of CLYBL and itaconate. Compared with PBS-treated controls, LPS stimulation led to a significant increase in CLYBL expression (Fig. 1J, K). Consistently, LC-MS analysis showed a notable decrease in intracellular itaconate and citramalyl-CoA levels following LPS treatment, which aligned with the results observed in peritoneal macrophages from CLP mice (Fig. 1L, M).

Collectively, these results suggest that CLYBL may play a crucial regulatory role in the immune response during sepsis-induced lung injury, particularly through its involvement in itaconate metabolism and modulation of macrophage function.

### Preparation and characterization of PEVs@PLGA@si-CLYBL nanoparticles

**Fig. 2:**
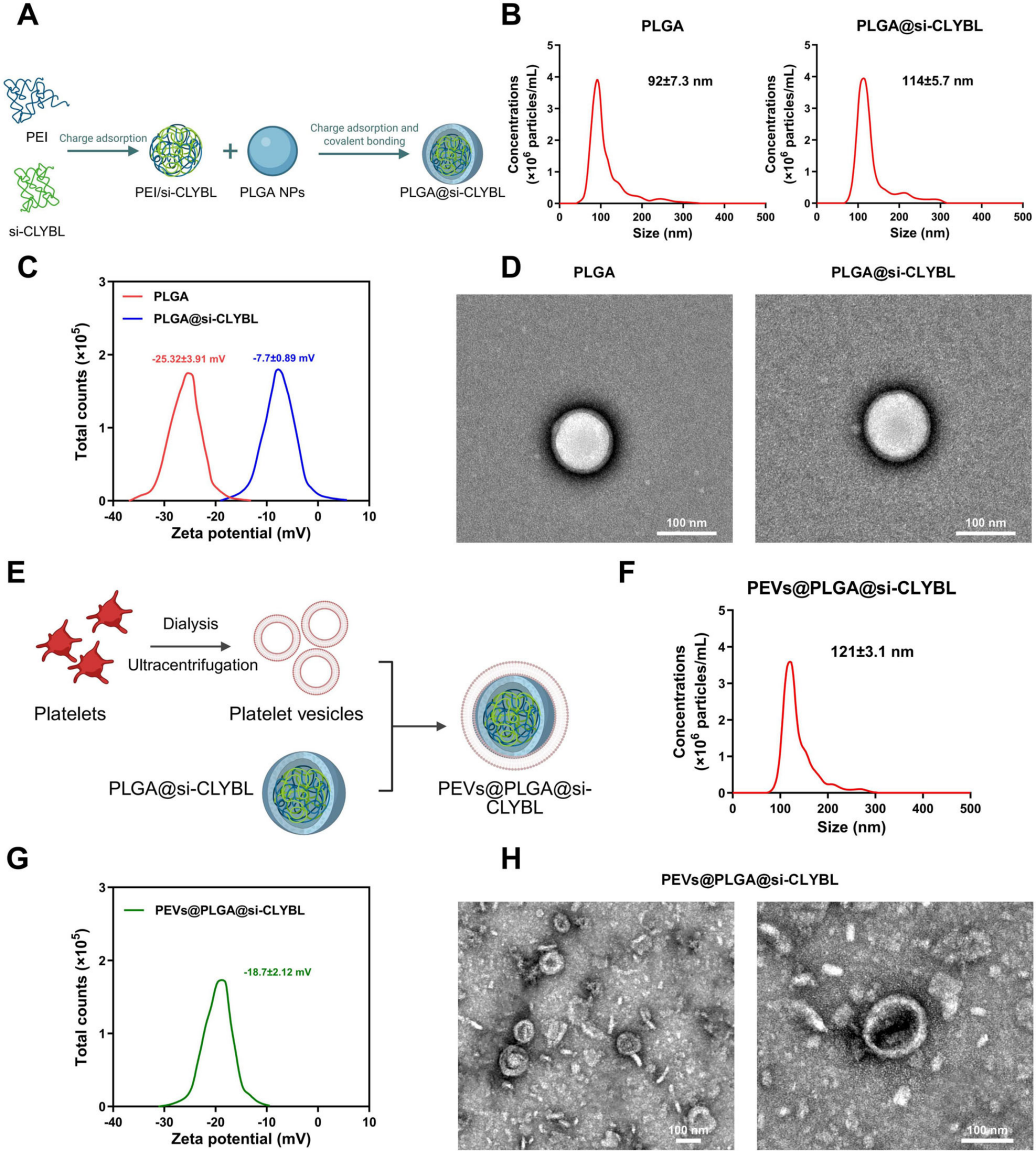
Fabrication and characterization of platelet membrane-derived hybrid nanoparticles. **A** Schematic illustration of the encapsulation process of PLGA@si-CLYBL nanoparticles (Created with BioRender.com); **B**, **C** DLS analysis of particle size and zeta potential for PLGA and PLGA@si-CLYBL nanoparticles; **D** TEM images showing the morphology of PLGA and PLGA@si-CLYBL nanoparticles (scale bar: 100 nm); **E** Schematic diagram of the preparation process for PEVs@PLGA@si-CLYBL nanoparticles; **F**, **G** DLS analysis of particle size and zeta potential of PEVs@PLGA@si-CLYBL nanoparticles; **H** TEM images of PEVs@PLGA@si-CLYBL nanoparticles showing spherical morphology and hybrid membrane coating (scale bar: 100 nm).

After confirming the significant upregulation of CLYBL in macrophages during sepsis-induced lung injury, we focused on developing a targeted delivery strategy for si-CLYBL using nanoparticles. To enhance the biocompatibility and targeting capability of si-CLYBL, we designed a hybrid nanoparticle system based on PEVs encapsulating polymeric nanoparticles. Initially, PLGA nanoparticles were synthesized via a self-assembly method, and si-CLYBL was loaded onto the particles via electrostatic adsorption, generating PLGA@si-CLYBL nanoparticles (Fig. 2A). DLS and TEM were used to characterize the size and morphology of the nanoparticles. DLS analysis revealed that both PLGA and PLGA@si-CLYBL nanoparticles had a uniform size distribution within the 100-200 nm range, with a negative zeta potential conducive to cellular uptake and biocompatibility (Fig. 2B, C). TEM images further confirmed their spherical and homogeneous morphology (Fig. 2D).

To improve delivery efficiency, PEVs were harvested from mouse blood via differential centrifugation and purified using dialysis and ultracentrifugation techniques (Fig. 2E). The purified PEVs were then fused with PLGA@si-CLYBL nanoparticles using a mild sonication method to form biomimetic hybrid nanoparticles, designated as PEVs@PLGA@si-CLYBL. DLS and TEM analyses showed that PEVs@PLGA@si-CLYBL nanoparticles exhibited slightly increased particle size, a membrane-like vesicular coating on the surface, and a reduced absolute zeta potential, indicating enhanced colloidal stability and improved cellular compatibility (Fig. 2F–H).

### Stability, release characteristics, and biocompatibility assessment of PEVs@PLGA@si-CLYBL nanoparticles

**Fig. 3:**
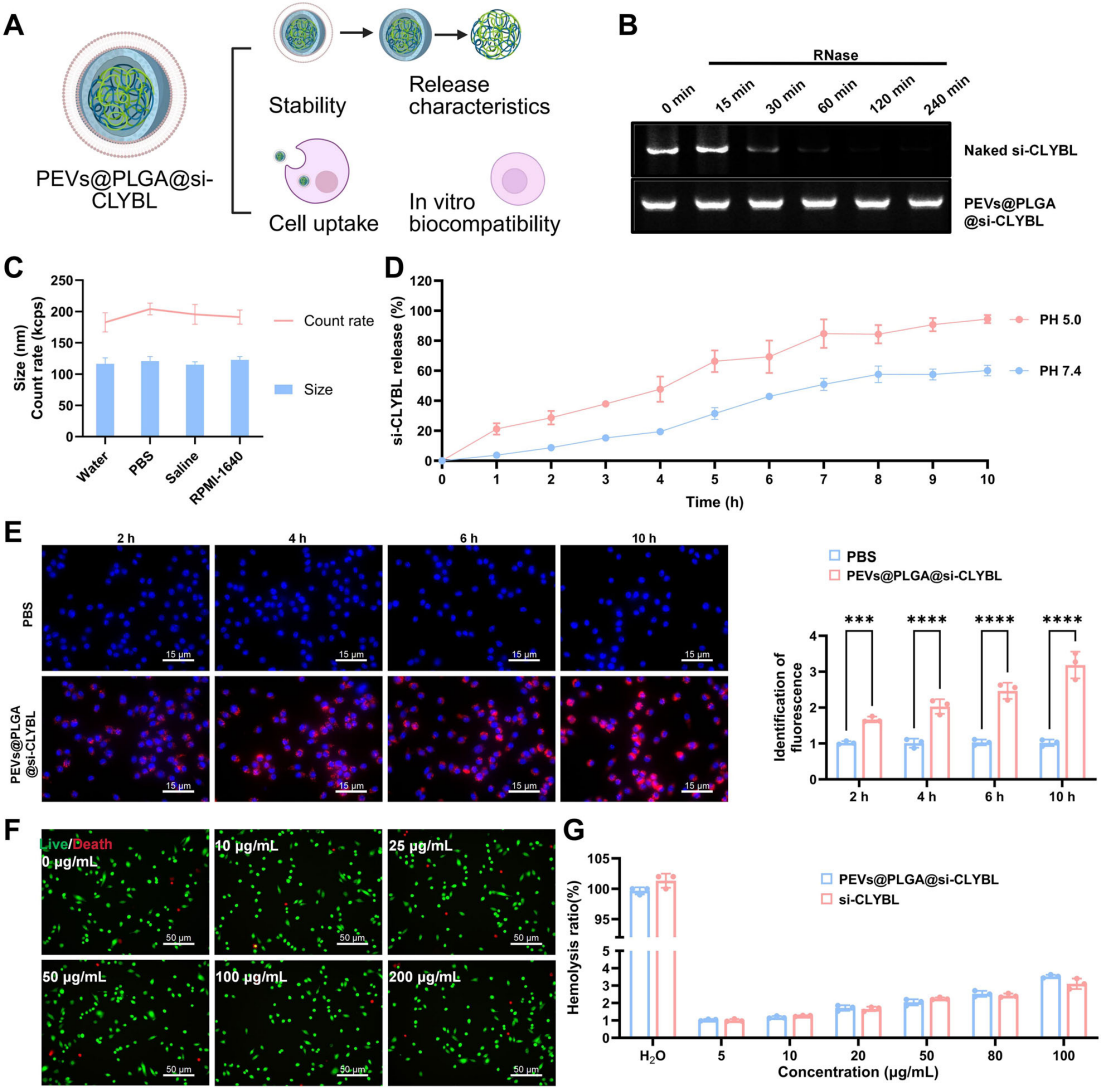
Stability, release characteristics, cellular uptake, cytotoxicity, and hemolytic assessment of PEVs@PLGA@si-CLYBL nanoparticles. **A** Schematic illustration of the stability evaluation process for PEVs@PLGA@si-CLYBL nanoparticles (Created with BioRender.com); **B** Agarose gel electrophoresis assessing the stability of naked si-CLYBL and PEVs@PLGA@si-CLYBL nanoparticles after incubation with RNase for 0, 15, 30, 60, 120, and 240 min; **C** Stability of PEVs@PLGA@si-CLYBL nanoparticles in various physiological media (ultrapure water, PBS, saline, and RPMI-1640); **D** siRNA release profiles of PEVs@PLGA@si-CLYBL under different pH conditions (pH 7.4, 6.5, and 5.0); **E** Fluorescence microscopy showing cellular uptake of PEVs@PLGA@si-CLYBL nanoparticles by macrophages (scale bar: 15 µm); **F** Live/dead cell staining to evaluate cytotoxicity of PEVs@PLGA@si-CLYBL toward macrophages (scale bar: 50 µm); **G** Hemolysis assay assessing the hemolytic activity of PEVs@PLGA@si-CLYBL nanoparticles on red blood cells. *p < 0.05, ****p < 0.0001.

We first evaluated the stability of PEVs@PLGA@si-CLYBL nanoparticles to determine their protective capacity in biological environments (Fig. 3A). Agarose gel electrophoresis was used to assess the stability of free si-CLYBL and PEVs@PLGA@si-CLYBL after incubation with RNase for different durations (0, 15, 30, 60, 120, and 240 min). The results demonstrated that, compared to free si-CLYBL, the nanoparticle formulation significantly delayed siRNA degradation, indicating its strong protective effect (Fig. 3B). Furthermore, the stability of PEVs@PLGA@si-CLYBL was assessed under various physiological conditions (water, PBS, saline, and RPMI-1640). The nanoparticles maintained consistent size and structure in all tested media, suggesting excellent stability under physiological conditions (Fig. 3C).

Next, we investigated the siRNA release behavior of PEVs@PLGA@si-CLYBL nanoparticles. In vitro release assays were conducted under simulated extracellular (pH 7.4) and endosomal (pH 5.0) environments. The results showed a significantly faster siRNA release at acidic pH, indicating pH-responsive release properties (Fig. 3D). Additionally, cellular uptake of rhodamine-labeled nanoparticles by macrophages was evaluated. Fluorescence microscopy revealed that PEVs@PLGA@si-CLYBL nanoparticles were efficiently internalized and evenly distributed within macrophages (Fig. 3E). These findings confirm that PEVs@PLGA@si-CLYBL nanoparticles exhibit excellent performance in terms of cellular uptake, stability, and controlled release behavior.

Furthermore, we assessed the cytotoxicity and biocompatibility of PEVs@PLGA@si-CLYBL nanoparticles. Live/dead cell staining assays were conducted to evaluate the cytotoxic effects on macrophages. The results showed that treatment with PEVs@PLGA@si-CLYBL did not significantly affect cell viability, with the majority of macrophages maintaining robust viability, indicating favorable biocompatibility (Fig. 3F). Hemolysis assays were subsequently performed to assess the biosafety of the nanoparticles in circulation. Even at higher concentrations, PEVs@PLGA@si-CLYBL nanoparticles did not induce noticeable hemolysis of red blood cells, further supporting their safety for in vivo applications (Fig. 3G).

Collectively, these findings indicate that PEVs@PLGA@si-CLYBL nanoparticles exhibit low cytotoxicity and excellent biocompatibility, making them suitable for systemic delivery. The nanoparticles demonstrated high stability, efficient siRNA protection, and excellent cellular uptake, with pH-responsive release that is enhanced under acidic conditions. The favorable safety profile further supports their potential for in vivo therapeutic use.

### PEVs@PLGA@si-CLYBL improves macrophage immune function by inhibiting itaconate depletion

**Fig. 4:**
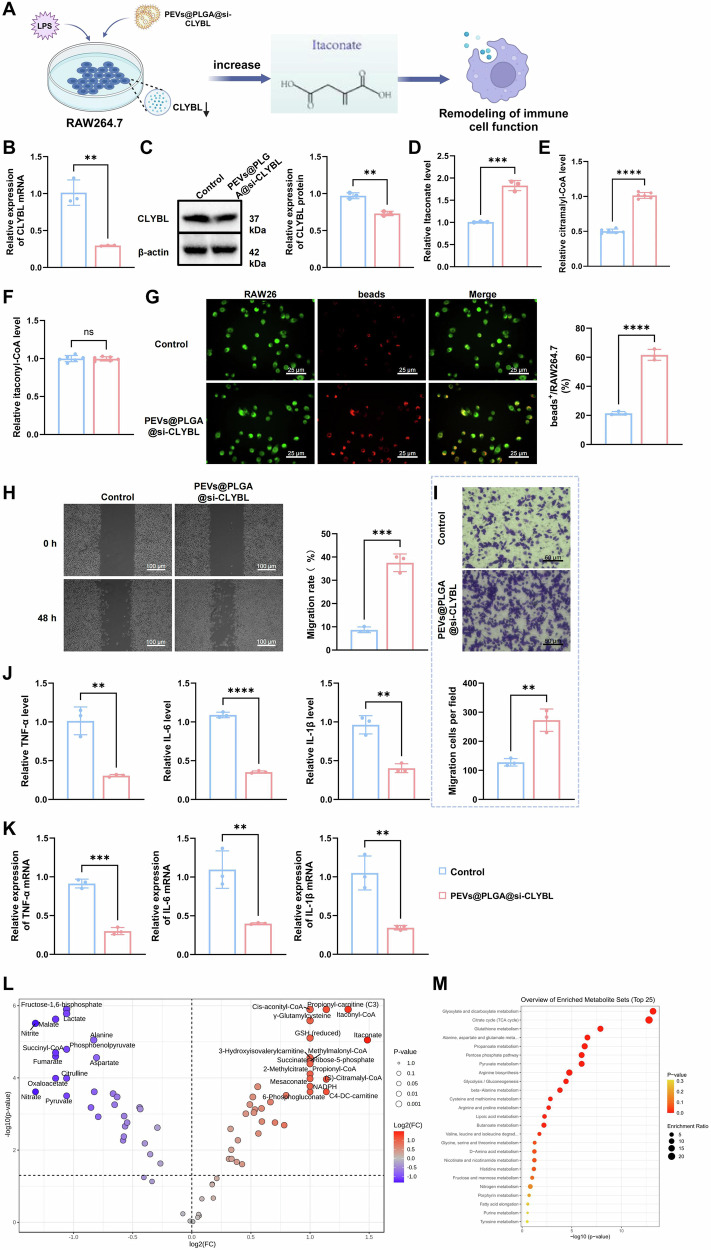
Immunomodulatory effects of PEVs@PLGA@si-CLYBL on macrophage function. **A** Schematic workflow illustrating the effect of PEVs@PLGA@si-CLYBL treatment on LPS-stimulated RAW264.7 macrophages (Created with BioRender.com); **B** RT-qPCR analysis of CLYBL mRNA expression in different groups of macrophages; **C** Western blot analysis of CLYBL protein expression; **D** High-resolution LC-MS quantification of intracellular itaconate levels; **E** LC-MS quantification of intracellular citramalyl-CoA levels; **F** LC-MS quantification of intracellular itaconyl-CoA levels; **G** Latex bead phagocytosis assay evaluating macrophage phagocytic capacity (scale bar: 20 µm); **H** Scratch assay assessing macrophage migration (scale bar: 100 µm); **I** Transwell assay evaluating macrophage chemotaxis (scale bar: 50 µm); **J** ELISA analysis of TNF-α, IL-6, and IL-1β secretion; **K** RT-qPCR analysis of TNF-α, IL-6, and IL-1β gene expression; **L** Volcano plot of differential metabolites between groups; **M** Pathway enrichment analysis of significantly altered metabolites. Experiments were performed in triplicate. *p < 0.05, **p < 0.01, ***p < 0.001, ****p < 0.0001; ns not significant.

We first investigated the impact of PEVs@PLGA@si-CLYBL on macrophage function using LPS-stimulated RAW264.7 murine macrophages (Fig. 4A). The experimental groups included a Control group (PBS-treated, LPS-stimulated RAW264.7 cells) and a PEVs@PLGA@si-CLYBL group (PEVs@PLGA@si-CLYBL-treated, LPS-stimulated RAW264.7 cells). RT-qPCR and Western blot analyses revealed that PEVs@PLGA@si-CLYBL significantly suppressed LPS-induced CLYBL expression, with both mRNA and protein levels of CLYBL markedly decreased compared to the Control group (Fig. 4B, C). These results suggest that PEVs@PLGA@si-CLYBL effectively downregulate LPS-induced CLYBL expression, laying the foundation for the subsequent reprogramming of immune cell function.

To further explore the mechanism, we measured intracellular itaconate and citramalyl-CoA levels via high-resolution LC-MS. The results demonstrated that treatment with PEVs@PLGA@si-CLYBL significantly increased itaconate and citramalyl-CoA levels in macrophages compared to the Control group, indicating that CLYBL inhibition may promote itaconate and citramalyl-CoA accumulation (Fig. 4D, E), but without affecting itaconyl-CoA level. Functionally, PEVs@PLGA@si-CLYBL treatment enhanced macrophage phagocytic activity, migratory capacity, and chemotactic response. Latex bead phagocytosis assays revealed a significant increase in phagocytic efficiency in the PEVs@PLGA@si-CLYBL group (Fig. 4G). Scratch wound assays showed that macrophages in the PEVs@PLGA@si-CLYBL group exhibited improved migratory ability (Fig. 4H), while Transwell assays indicated enhanced chemotaxis (Fig. 4I). Moreover, ELISA and RT-qPCR analyses revealed that treatment with PEVs@PLGA@si-CLYBL significantly suppressed the expression of proinflammatory cytokines TNF-α, IL-6, and IL-1β in LPS-treated cells (Fig. 4J, K). Collectively, these results indicate that PEVs@PLGA@si-CLYBL effectively attenuate M1 macrophage hyperactivation and facilitate functional reprogramming of immune cells.

To determine whether CLYBL silencing exerts its effects solely by promoting itaconate accumulation or also induces broader metabolic reprogramming, we performed semi-targeted metabolomics profiling in LPS-stimulated RAW264.7 macrophages (Control group) and those additionally treated with PEVs@PLGA@si-CLYBL (Treatment group). Volcano plot analysis revealed that, in addition to the marked elevation of itaconate and its intermediates—(S)-citramalyl-CoA and itaconyl-CoA—several metabolites involved in propionate metabolism, including propionyl-CoA, propionyl-carnitine (C3), methylmalonyl-CoA, and 2-methylcitrate, were significantly increased. Concurrently, metabolites linked to glutathione metabolism, such as γ-glutamylcysteine and reduced GSH, were also elevated. In contrast, downstream TCA cycle intermediates (fumarate and succinyl-CoA), and energy metabolism–related metabolites (pyruvate, phosphoenolpyruvate, and lactate) were markedly decreased (Fig. 4L). Pathway enrichment analysis further demonstrated significant enrichment not only in “itaconate degradation” but also in the “citrate cycle (TCA cycle),” “propionate metabolism,” and “glutathione metabolism” pathways. Additionally, pathways associated with redox regulation and metabolic rerouting, including glyoxylate and dicarboxylate metabolism and the pentose phosphate pathway, were also affected (Fig. 4M).

Collectively, these findings indicate that CLYBL silencing not only enhances the accumulation of itaconate and its upstream intermediates but also induces propionate metabolic blockade, redistribution of TCA cycle intermediates, and activation of the glutathione-NADPH redox system. This multi-layered metabolic reprogramming suggests that CLYBL depletion reinforces immune homeostasis in macrophages through coordinated metabolic regulation, with itaconate accumulation serving as a central regulatory node.

CLYBL participates not only in the itaconate degradation pathway but also in propionate metabolism [20, 21]. To determine whether CLYBL modulates macrophage immune function primarily through regulating itaconate metabolism, we stimulated RAW264.7 murine macrophages with LPS and examined the expression of SUCLG1—one of the enzymes required for the first step of the itaconate degradation pathway (itaconate → itaconyl-CoA) [29, 30]. Our results showed that SUCLG1 mRNA and protein levels were markedly upregulated following LPS stimulation (Fig. S2A, B). We next silenced SUCLG1 using siRNA to evaluate its functional impact on LPS-treated macrophages. Compared with the Control group, siSUCLG1 efficiently reduced SUCLG1 expression at both the mRNA and protein levels (Fig. S2C, D), confirming successful knockdown and providing a basis for subsequent assessment of macrophage functional reprogramming. High-resolution LC–MS analysis further revealed a significant accumulation of itaconate in siSUCLG1-treated macrophages, indicating that SUCLG1 inhibition promotes itaconate buildup (Fig. S2E). Functionally, macrophages in the siSUCLG1 group exhibited markedly enhanced phagocytosis, migration, and chemotaxis compared with the Control group. The latex bead engulfment assay demonstrated significantly improved phagocytic activity following SUCLG1 knockdown (Fig. S2F). Consistently, scratch wound assays showed enhanced migratory capacity (Fig. S2G), while Transwell assays revealed increased chemotaxis (Fig. S2H). Moreover, ELISA and RT-qPCR analyses showed that siSUCLG1 significantly reduced LPS-induced expression of proinflammatory cytokines TNF-α, IL-6, and IL-1β (Fig. S2I, J). Collectively, these findings demonstrate that SUCLG1 silencing effectively promotes itaconate accumulation, thereby suppressing excessive M1 macrophage activation and facilitating immune functional reprogramming. These results support the conclusion that the beneficial effects of CLYBL depletion in sepsis are primarily mediated by increased itaconate accumulation rather than alterations in other metabolic pathways.

We further conducted rescue experiments to assess the immunomodulatory effects of PEVs@PLGA@si-CLYBL on LPS-stimulated macrophages and to investigate the role of CLYBL overexpression. RAW264.7 murine macrophages were treated with either PEVs@PLGA@si-CLYBL alone (PEVs@PLGA@si-CLYBL group) or in combination with CLYBL overexpression (PEVs@PLGA@si-CLYBL + oe-CLYBL group). RT-qPCR and Western blot analyses confirmed that CLYBL expression was significantly downregulated in the PEVs@PLGA@si-CLYBL group, while it was markedly upregulated in the PEVs@PLGA@si-CLYBL + oe-CLYBL group, confirming successful gene overexpression (Fig. S3A, B).

Next, LC-MS analysis was performed to evaluate itaconate accumulation. Itaconate levels were significantly increased in the PEVs@PLGA@si-CLYBL group, whereas the PEVs@PLGA@si-CLYBL + oe-CLYBL group exhibited a notable reduction in itaconate accumulation, suggesting that CLYBL overexpression partially inhibited itaconate buildup (Fig. S3C). Functional assays revealed that the PEVs@PLGA@si-CLYBL + oe-CLYBL group exhibited enhanced phagocytic activity, migratory capacity, and chemotactic response compared to the PEVs@PLGA@si-CLYBL group (Fig. S3D–F). Moreover, ELISA and RT-qPCR analyses showed that expression levels of proinflammatory cytokines, including TNF-α, IL-6, and IL-1β, were significantly elevated in the overexpression group (Fig. S3G, H), indicating that CLYBL overexpression may enhance macrophage activation and amplify inflammatory responses.

### PEVs@PLGA@si-CLYBL promotes alveolar epithelial repair by reprogramming macrophage function

**Fig. 5:**
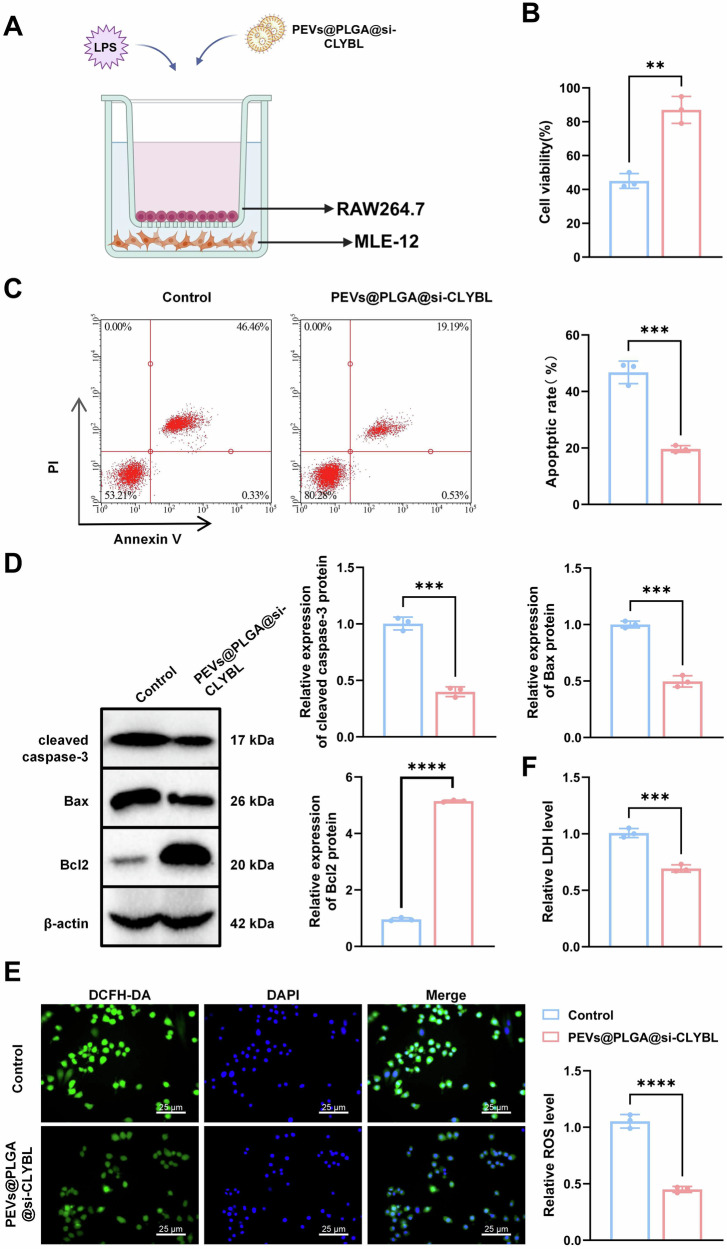
Effects of PEVs@PLGA@si-CLYBL on a co-culture model of LPS-treated macrophages and lung epithelial cells. **A** Schematic diagram of the experimental design for the macrophage–epithelial cell co-culture model (Created with BioRender.com); **B** CCK-8 assay evaluating the effects of PEVs@PLGA@si-CLYBL on cell proliferation and viability; **C** Flow cytometry analysis of macrophage apoptosis following treatment; **D** Western blot analysis of apoptosis-related proteins (cleaved caspase-3, Bcl2, Bax) in macrophages; **E** ROS detection assay assessing intracellular ROS levels in epithelial cells (scale bar: 25 µm); **F** LDH release assay evaluating the membrane-stabilizing effects of PEVs@PLGA@si-CLYBL. Experiments were repeated three times. *p < 0.05, **p < 0.01, ***p < 0.001, ****p < 0.0001.

We first evaluated the effects of PEVs@PLGA@si-CLYBL in a co-culture system of LPS-stimulated RAW264.7 murine macrophages and MLE-12 murine alveolar epithelial cells (Fig. 5A). Cell proliferation and viability were assessed using the CCK-8 assay. Results showed that PEVs@PLGA@si-CLYBL significantly enhanced cell viability and proliferation in the co-culture system. Compared with the Control group, the PEVs@PLGA@si-CLYBL group exhibited markedly improved cellular activity (Fig. 5B). Flow cytometry analysis revealed that PEVs@PLGA@si-CLYBL significantly reduced apoptosis in macrophages, suggesting that it may suppress macrophage overactivation and protect against cell death (Fig. 5C). This was further confirmed by Western blot analysis, which showed downregulation of cleaved caspase-3 and Bax, and upregulation of the anti-apoptotic protein Bcl2 (Fig. 5D).

Given the critical role of oxidative stress in lung injury, we assessed ROS levels and found that PEVs@PLGA@si-CLYBL treatment significantly reduced intracellular ROS in both macrophages and epithelial cells, indicating an antioxidative effect (Fig. 5E). Additionally, LDH release assays demonstrated that PEVs@PLGA@si-CLYBL markedly decreased extracellular LDH levels, further supporting its protective role in maintaining cell membrane integrity (Fig. 5F). These findings indicate that PEVs@PLGA@si-CLYBL facilitate epithelial cell repair and survival by mitigating M1 macrophage overactivation and oxidative stress.

To investigate the impact of CLYBL overexpression on macrophages and alveolar epithelial cells treated with PEVs@PLGA@si-CLYBL, a rescue experiment was designed by comparing the PEVs@PLGA@si-CLYBL group with the PEVs@PLGA@si-CLYBL + oe-CLYBL group. First, cell proliferation and viability were evaluated using the CCK-8 assay. The results showed that the PEVs@PLGA@si-CLYBL group exhibited significantly higher cell viability and proliferation compared to the PEVs@PLGA@si-CLYBL + oe-CLYBL group, suggesting that CLYBL overexpression may counteract the protective effects of PEVs@PLGA@si-CLYBL (Fig. S4A). Flow cytometry analysis further demonstrated a significantly increased apoptosis rate in both macrophages and epithelial cells in the PEVs@PLGA@si-CLYBL + oe-CLYBL group (Fig. S4B). Western blot analysis revealed that cleaved caspase-3 and Bax levels were markedly upregulated, whereas the anti-apoptotic protein Bcl2 was downregulated in the CLYBL overexpression group (Fig. S4C), indicating enhanced apoptosis.

Regarding oxidative stress, ROS analysis showed markedly elevated intracellular ROS levels in the CLYBL overexpression group, supporting the notion that CLYBL overexpression exacerbates oxidative damage (Fig. S4D). The LDH release assay also indicated significantly increased LDH levels in the PEVs@PLGA@si-CLYBL + oe-CLYBL group compared to the PEVs@PLGA@si-CLYBL group, suggesting enhanced membrane damage (Fig. S4E).

Collectively, these findings suggest that CLYBL overexpression reverses the protective effects of PEVs@PLGA@si-CLYBL, resulting in reduced cell viability, increased apoptosis, and elevated oxidative stress in both macrophages and alveolar epithelial cells.

### PEVs@PLGA@si-CLYBL nanoparticles promote lung repair in sepsis via immune modulation

**Fig. 6:**
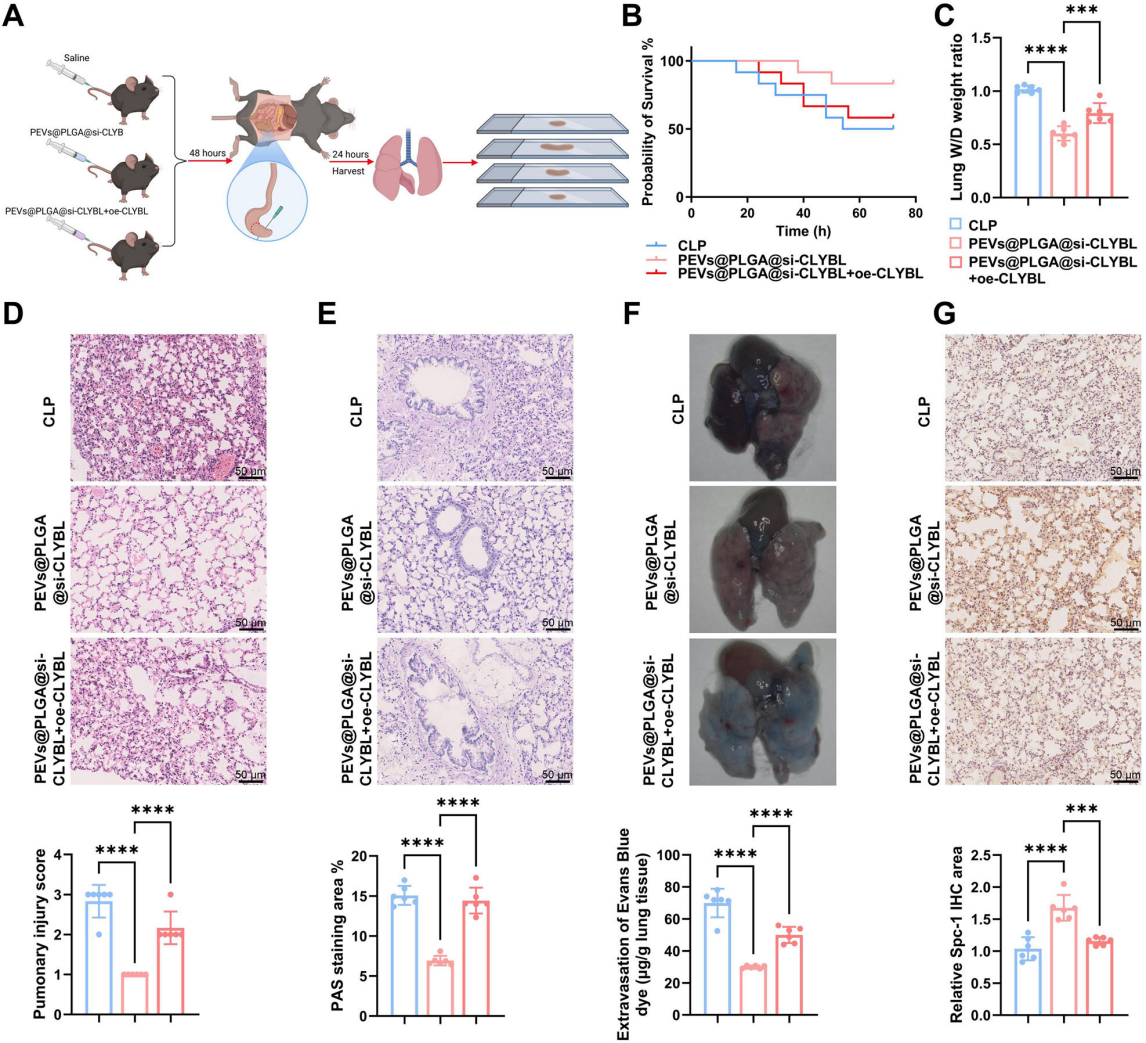
Therapeutic effects of PEVs@PLGA@si-CLYBL nanoparticles on lung injury in a sepsis mouse model. **A** Schematic illustration of the experimental workflow for treating septic mice with PEVs@PLGA@si-CLYBL nanoparticles (Created with BioRender.com); **B** Kaplan–Meier survival curve comparing the survival rates of CLP and PEVs@PLGA@si-CLYBL-treated mice; **C** Lung W/D weight ratio indicating pulmonary edema severity; **D**–**F** H&E staining, PAS staining, and Evans blue staining to evaluate histopathological damage and vascular permeability in lung tissue (scale bar: 50 µm); **G** IHC analysis of Spc-1 expression in lung tissue (scale bar: 50 µm). Each group included 6 mice. *p < 0.05, ***p < 0.001, ****p < 0.0001.

To further evaluate the therapeutic effects of PEVs@PLGA@si-CLYBL nanoparticles on sepsis-induced lung injury, we conducted a comprehensive analysis including histopathological assessment, immune cell functional remodeling, and inflammation profiling (Fig. 6A). Western blot and RT-qPCR results showed that CLYBL expression in lung tissues was significantly reduced in the PEVs@PLGA@si-CLYBL-treated group compared to the CLP group, whereas CLYBL expression was significantly elevated in the PEVs@PLGA@si-CLYBL + oe-CLYBL group (Fig. S5A, B). Survival analysis indicated that PEVs@PLGA@si-CLYBL treatment significantly improved survival in septic mice, while CLYBL overexpression notably abrogated this benefit (Fig. 6B). Measurement of pulmonary edema revealed a significantly lower lung W/D weight ratio in the PEVs@PLGA@si-CLYBL group, suggesting reduced pulmonary edema. In contrast, CLYBL overexpression reversed this effect (Fig. 6C).

Histological analyses, including H&E, PAS, and Evans blue staining, demonstrated that PEVs@PLGA@si-CLYBL treatment substantially attenuated lung injury, restored alveolar architecture, and reduced vascular permeability. These improvements were abolished upon CLYBL overexpression (Fig. 6D–F). IHC further revealed that Spc-1 expression was markedly increased in the PEVs@PLGA@si-CLYBL group, indicating enhanced alveolar epithelial repair. However, this effect was suppressed in the presence of CLYBL overexpression (Fig. 6G).

**Fig. 7:**
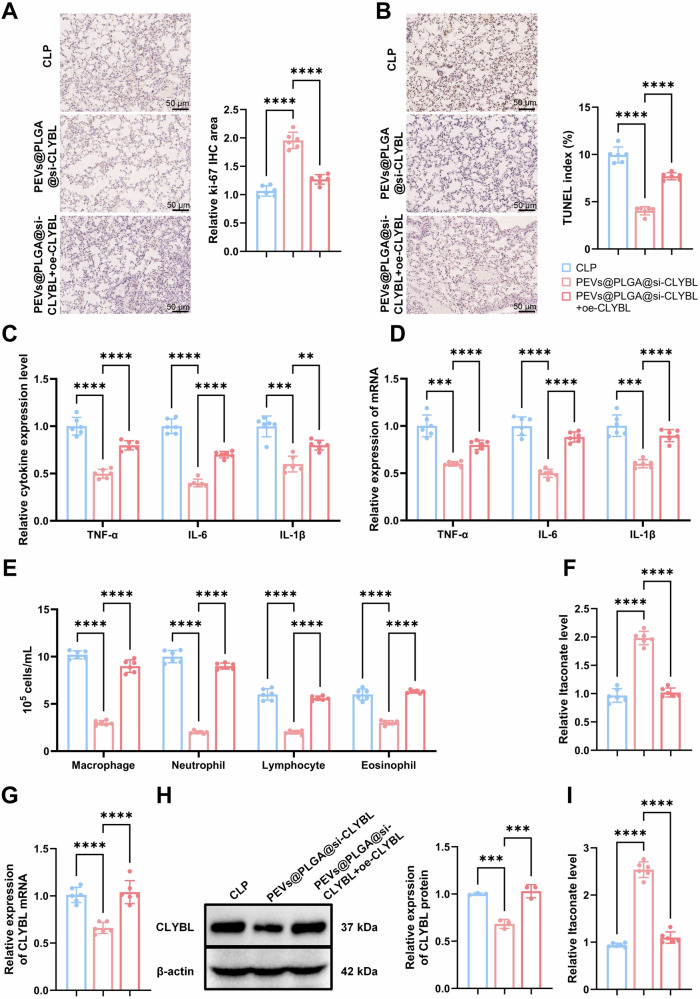
PEVs@PLGA@si-CLYBL nanoparticles modulate immune responses and inflammatory cell function in septic mice. **A** Ki-67 staining to assess lung epithelial cell proliferation (scale bar: 50 μm); **B** TUNEL staining to evaluate epithelial cell apoptosis (scale bar: 50 μm); **C**, **D** ELISA and RT-qPCR analysis of TNF-α, IL-6, and IL-1β levels in BALF; **E** Immune cell profiling of inflammatory cell proportions in BALF; **F** LC-MS analysis of itaconate levels in lung tissue; **G**, **H** Western blot and RT-qPCR analysis of CLYBL expression in peritoneal macrophages; **I** LC-MS measurement of itaconate accumulation in peritoneal macrophages. All experiments were repeated three times with six animals per group. *p < 0.05, **p < 0.01, ***p < 0.001, ****p < 0.0001.

Furthermore, Ki-67 staining revealed a marked increase in alveolar epithelial cell proliferation in the PEVs@PLGA@si-CLYBL-treated mice (Fig. 7A), while TUNEL staining showed a significant reduction in apoptosis (Fig. 7B). In contrast, CLYBL overexpression promoted cell apoptosis. ELISA and RT-qPCR analyses demonstrated that the levels of TNF-α, IL-6, and IL-1β in BALF were significantly decreased in the PEVs@PLGA@si-CLYBL group compared to the CLP group (Fig. 7C, D), indicating a robust suppression of the inflammatory response. This anti-inflammatory effect was reversed by CLYBL overexpression. Further immune cell profiling revealed that the proportions of various inflammatory cells in the BALF were significantly reduced following PEVs@PLGA@si-CLYBL treatment (Fig. 7E), particularly neutrophils and macrophages, suggesting that this treatment may alleviate sepsis-induced inflammation by modulating immune cell activity. Conversely, CLYBL overexpression led to an increased infiltration of inflammatory cells. In addition, the level of itaconate in lung tissue was significantly elevated following treatment (Fig. 7F). Peritoneal macrophages were also isolated from mice in each group. Compared to the CLP group, the PEVs@PLGA@si-CLYBL group showed reduced expression of CLYBL at both mRNA and protein levels, accompanied by a notable accumulation of itaconate. However, CLYBL overexpression suppressed this accumulation (Fig. 7G–I).

Collectively, these findings suggest that PEVs@PLGA@si-CLYBL nanoparticles effectively alleviate sepsis-induced lung injury and promote alveolar epithelial repair by remodeling immune cell function and regulating itaconate accumulation.

### Biosafety evaluation of PEVs@PLGA@si-CLYBL nanoparticles in the treatment of sepsis-induced lung injury

We conducted a series of experiments to evaluate the biosafety of PEVs@PLGA@si-CLYBL nanoparticles. First, histopathological examinations were performed on major organs (heart, liver, spleen, and kidneys) from mice in each group. H&E staining revealed no significant histological abnormalities or toxic effects in the PEVs@PLGA@si-CLYBL group, with tissue structures comparable to those of normal mice (Fig. S6A). To further assess systemic biosafety, serum biochemical parameters reflecting organ function were measured using an automated biochemical analyzer. The results showed that serum markers in the PEVs@PLGA@si-CLYBL-treated mice remained within normal ranges, with no evidence of organ dysfunction (Fig. S6B).

These findings collectively support the favorable biocompatibility and systemic safety of PEVs@PLGA@si-CLYBL nanoparticles, suggesting that their therapeutic application in sepsis does not cause significant organ toxicity in vivo.

### Transcriptomic analysis reveals that PEVs@PLGA@si-CLYBL regulate immune cell infiltration and metabolic reprogramming to repair sepsis-induced lung injury

**Fig. 8:**
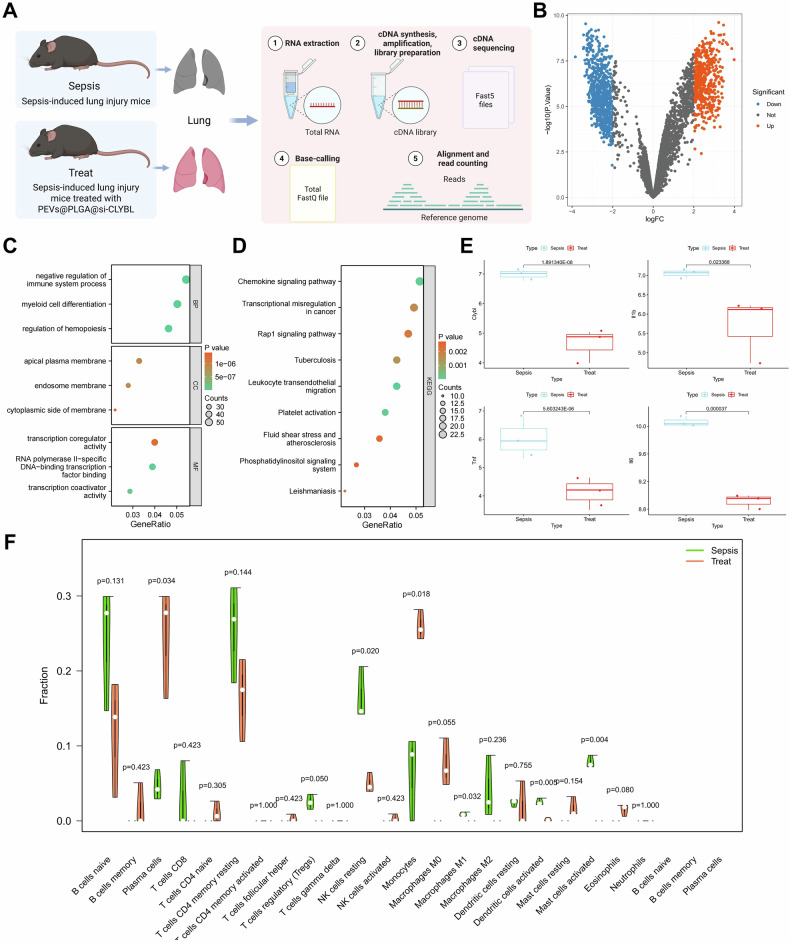
Transcriptomic analysis reveals the regulatory effects of PEVs@PLGA@si-CLYBL on sepsis-induced lung injury. **A** Schematic of the transcriptome sequencing strategy (Created with BioRender.com); **B** Volcano plot showing DEGs in lung tissues between the PEVs@PLGA@si-CLYBL-treated and CLP groups; **C** GO enrichment analysis of DEGs; **D** KEGG pathway enrichment analysis; **E** Expression profiles of CLYBL, IL-1β, IL-6, and TNF-α; **F** Violin plot of immune cell composition differences between sepsis and treatment groups. Sepsis: *n* = 3; Treatment: *n* = 3.

In this study, total RNA was successfully extracted from lung tissues of the CLP group (mice subjected to CLP-induced sepsis) and the PEVs@PLGA@si-CLYBL treatment group (CLP mice intravenously injected with PEVs@PLGA@si-CLYBL at 0.6 mg/kg). High-throughput RNA sequencing was then performed to analyze the transcriptomic changes (Fig. 8A). After data preprocessing and differential gene expression analysis, a total of 1,157 significantly altered genes were identified in the PEVs@PLGA@si-CLYBL group compared with the CLP group, including 484 upregulated and 673 downregulated genes (Fig. 8B). GO and KEGG pathway enrichment analyses revealed that these DEGs were predominantly enriched in immune responses, inflammatory processes, and cell cycle regulation (Fig. 8C, D), which was consistent with the results of GSEA enrichment analysis (Fig. S7). Notably, key immune-related genes such as IL-1β, IL-6, and TNF-α were significantly downregulated in the PEVs@PLGA@si-CLYBL group, along with CLYBL (Fig. 8E).

Immune cell infiltration analysis revealed a significant reduction in M1 macrophages following treatment, whereas M2 macrophage levels remained unchanged, indicating a functional repolarization of macrophages. The increase in monocyte proportions, together with the observed accumulation of itaconate, suggested that macrophages may undergo non-classical activation driven by metabolic regulation. In addition to macrophages, plasma cells were markedly elevated, and a shift in B cells from naive to memory phenotype was observed, indicating enhanced humoral immunity and immune maturation. Dendritic cell activation was suppressed, and the proportion of resting mast cells was reduced, reflecting an overall attenuation of inflammatory responses. Collectively, these findings suggest that CLYBL-targeted intervention not only reprograms macrophage function but also promotes a shift in the pulmonary immune microenvironment from an inflammatory state toward homeostasis (Figs. 8F and S8).

## Discussion

This study focused on the therapeutic strategy for sepsis-induced lung injury by designing and evaluating an innovative siRNA delivery system—PEVs@PLGA@si-CLYBL. The goal was to precisely modulate macrophage immunometabolic status and thereby ameliorate sepsis-induced pulmonary inflammation. We demonstrated that this delivery system not only possesses favorable lung-targeting capability and biocompatibility in vivo but also effectively silences CLYBL, leading to the accumulation of itaconate, functional reprogramming of macrophages, and accelerated repair of alveolar epithelial cells. This strategy presents a novel therapeutic perspective, encompassing delivery system design, target selection, and mechanistic validation, which sets it apart from conventional anti-inflammatory or broadly immunosuppressive approaches. It highlights strong theoretical innovation and translational potential.

CLYBL, a mitochondrial citrate lyase-like protein, has been primarily studied in metabolic contexts; however, its role in immune cells—especially macrophages—has only recently gained attention [23, 31, 32]. Previous studies suggest that CLYBL regulates the citrate metabolic shunt, influencing downstream itaconate synthesis and thereby modulating immune response states [23]. Building on this foundation, our study is the first to systematically investigate the expression pattern and functional significance of CLYBL in a sepsis-induced lung injury model. Our data show that CLYBL is significantly upregulated in lung tissue and peritoneal macrophages of CLP mice, indicating its increased activity in sepsis-related inflammatory responses. These findings are consistent with Shen et al.‘s reports on the link between CLYBL and itaconate metabolism; however, our work further identifies CLYBL as a key node in immunometabolic regulation, thereby broadening its relevance in inflammatory disease research.

Itaconate, an immunometabolite derived from the tricarboxylic acid cycle, has recently been widely recognized for its critical role in regulating macrophage inflammatory states [33, 34]. It exerts immunosuppressive and tissue-protective effects through multiple mechanisms, including inhibition of succinate dehydrogenase activity, activation of the Nrf2 signaling pathway, and suppression of pro-inflammatory cytokine expression [35, 36]. Most previous studies have focused on the exogenous supplementation of itaconate or its derivatives to modulate inflammation. In contrast, our study targeted endogenous metabolic regulation by silencing CLYBL to elevate intracellular itaconate levels in macrophages, thereby promoting their phenotypic reprogramming. This strategy more closely mimics physiological metabolic regulation, enabling more precise and controllable immune intervention and potentially improving therapeutic efficacy and safety.

Regarding the design of the delivery system, we employed PLGA as the core carrier for siRNA and incorporated PEVs to construct PEVs@PLGA@si-CLYBL, which exhibits enhanced targeting and delivery efficiency. Compared to traditional carriers such as cationic liposomes and PEI, PLGA offers superior biodegradability and encapsulation efficiency, while PEVs provide immune evasion and tissue-targeting capabilities due to their self-derived membrane-like properties [37]. Our in vitro cellular uptake, drug release assays, and in vivo biodistribution studies confirmed the effective pulmonary accumulation and sustained release profile of this system. The vesicle-fusion-based delivery platform remains a novel approach in the current field of siRNA therapeutics, holding significant potential for future applications.

In terms of functionality, our in vitro and in vivo experiments further confirmed that PEVs@PLGA@si-CLYBL nanoparticles effectively silenced CLYBL expression, significantly increased intracellular itaconate levels in macrophages, inhibited excessive M1 polarization, and promoted lung epithelial cell repair. This coordinated mechanism of “immunometabolic modulation-inflammation resolution-tissue regeneration” offers novel insights into the pathophysiology of sepsis-induced lung injury. Although the overexpression efficiency of adenoviral oe-CLYBL was relatively low—likely due to individual variability among animals, limited viral infectivity caused by lung tissue injury and immune-cell infiltration in the sepsis model, or the possibility that sample collection at 24 h post-infection did not coincide with the peak of protein expression—the pro-inflammatory effects observed upon CLYBL overexpression nevertheless provide inverse evidence supporting CLYBL knockdown as an effective therapeutic target in sepsis.

Additionally, transcriptomic analysis revealed that this intervention strategy significantly regulated key signaling pathways associated with oxidative stress, lipid metabolism, and cell migration, providing molecular-level evidence of its systemic effects. This multi-dimensional mechanistic validation further enhances the scientific rigor and credibility of our findings. No obvious toxic effects were detected in vivo, as shown by normal liver and kidney function, blood parameters, and histopathology, indicating the favorable biosafety of the PEVs@PLGA@si-CLYBL system. By leveraging PEVs as natural carriers, this delivery strategy minimizes immune responses and offers better translational potential compared to synthetic materials. The platform is also adaptable to other inflammation-related targets. In summary, this study developed a novel PEVs@PLGA@si-CLYBL nanodelivery system targeting the CLYBL/itaconate metabolic axis in sepsis-induced lung injury. The system effectively regulated macrophage immunometabolism and promoted tissue repair, advancing both therapeutic target selection and delivery design. Despite its efficacy and safety in animal models, challenges remain for clinical translation, including standardization and storage stability. Future research should address long-term biosafety, optimize production, and expand preclinical testing, paving the way for broader application in immune-related diseases.

## Conclusion

This study demonstrates the use of PEVs@PLGA@si-CLYBL in the treatment of sepsis-induced lung injury. By effectively silencing CLYBL expression, the nanoparticles significantly promote the accumulation of itaconate in macrophages, leading to the reprogramming of immune cell function. This immunomodulation, in turn, attenuates the inflammatory response and facilitates the repair of alveolar epithelial cells, thereby markedly improving pulmonary injury caused by sepsis. Transcriptomic analysis further revealed that the therapeutic efficacy of this nanoplatform is mediated through the optimization of immune responses and cellular repair pathways. In vivo biosafety evaluation confirmed the excellent biocompatibility and lack of significant toxicity of the nanoparticles, supporting their clinical translational potential for immune-targeted therapies in sepsis-induced lung injury.

Collectively, this work proposes an innovative immunoregulatory strategy for treating sepsis-related lung injury. By enhancing endogenous itaconate accumulation and promoting immune cell reprogramming, PEVs@PLGA@si-CLYBL nanoparticles offer a novel approach to facilitate lung tissue repair, representing significant scientific value. Clinically, this strategy holds promise for mitigating pulmonary damage in sepsis patients and may serve as a critical component in the development of future immunotherapeutic interventions.

## Materials and methods

### Ethics

All animal experiments were conducted in strict accordance with relevant ethical guidelines and regulations. All procedures were approved by the Institutional Animal Care and Use Committee (IACUC) (Ethical Approval Number: 2023-123-01). Animals were housed and cared for in a humane environment, with every effort made to minimize their suffering. At the end of the experiments, all mice were humanely euthanized under ether anesthesia.

### Establishment of a sepsis-induced lung injury mouse model

To establish a mouse model of sepsis-induced lung injury, 8-week-old C57BL/6 mice (*n* = 8 per group; purchased from Beijing Vital River Laboratory Animal Technology Co., Ltd., China) were randomly assigned to experimental groups. Mice were anesthetized with 1% (w/v) sodium pentobarbital (11715, Sigma-Aldrich, USA), and a 1-2 cm midline abdominal incision was made to expose the cecum. Sepsis was induced via CLP, where a portion of the cecum was ligated and punctured before being returned to the peritoneal cavity, and the abdominal wall was sutured. In the sham-operated group, only the abdominal incision was made without cecal ligation. At the end of surgery, all mice received an intraperitoneal injection of 0.9% saline for fluid resuscitation and were allowed free access to food and water. Postoperative monitoring was conducted over 24 h to ensure a smooth recovery. Mice were maintained under a 12-h light/dark cycle at 22 ± 2 °C and 60-70% relative humidity, with ad libitum access to water and standard chow. On day 2 before CLP, mice received a single intravenous injection of PEVs@PLGA@si-CLYBL nanoparticles (0.6 mg/kg) and oe-CLYBL lentivirus (200 µL, 1×10⁸ TU), followed by another injection before surgery as part of the intervention treatment [38, 39].

In Vivo Biosafety Evaluation: To assess overall health, body weight changes were monitored throughout the experiment. At the end of the study, the heart, liver, spleen, and kidneys were harvested for histological examination using hematoxylin and eosin (H&E) staining to evaluate tissue morphology. Blood samples were collected for serum biochemical analysis using an automated clinical chemistry analyzer (Hitachi 7180, Japan) to measure creatine kinase (CK), lactate dehydrogenase (LDH), alanine aminotransferase (ALT), aspartate aminotransferase (AST), creatinine (Cr), and urea levels for liver and kidney function assessment.

### Lung edema assessment

Lung edema was evaluated by measuring the wet-to-dry (W/D) weight ratio of lung tissues. Briefly, mouse lung tissues were excised and weighed immediately to obtain the wet weight. The tissues were then dried in a 65 °C oven for 48 h to determine the dry weight. The severity of pulmonary edema was quantified by calculating the W/D weight ratio, with an increased ratio indicating the presence of edema.

### Evans blue staining

Evans blue staining was performed to evaluate pulmonary vascular permeability. A 2% Evans blue solution (E2129, Sigma-Aldrich, USA) was injected via the tail vein at a dose of 4 ml/kg. Three h after injection, mice were perfused transcardially with phosphate-buffered saline (PBS) to remove intravascular dye. Mice were then euthanized, and their lung tissues were harvested and homogenized in 1 mL of N, N-dimethylformamide (822275, Sigma-Aldrich, USA). The homogenates were incubated in a 55 °C water bath overnight and centrifuged at 12,000 × *g* for 20 min. The absorbance of the supernatant was measured at 620 nm using a spectrophotometer (Thermo Scientific, USA). Pulmonary vascular permeability was calculated based on Evans blue extravasation using the formula: Evans blue extravasation (μg/g-1) = [Evans blue concentration (μg/ml-1) × 1 ml] / tissue wet weight (g).

### Histological and immunohistochemical analyses

Lung tissues were fixed in 4% paraformaldehyde, dehydrated, paraffin-embedded, and sectioned at 4 μm thickness for various staining procedures. Hematoxylin and eosin (H&E) staining was performed to evaluate histopathological changes and scored for alveolar edema, hemorrhage, leukocyte infiltration, and alveolar wall thickness by blinded observers. Periodic acid-Schiff (PAS) staining assessed glycogen and glycoprotein accumulation in alveolar epithelial cells. For immunohistochemistry (IHC), sections were incubated overnight at 4 °C with primary antibodies, including surfactant protein C and Ki-67, followed by HRP-conjugated secondary antibody and DAB visualization. Apoptosis in lung tissues was detected using a TUNEL assay kit; stained sections were visualized microscopically, and the apoptotic index was calculated as the percentage of TUNEL-positive cells. All analyses were performed on randomly selected fields under a light microscope to ensure objective and reproducible results.

### BALF collection and cytological analysis

Bronchoalveolar lavage fluid (BALF) was collected by tracheal cannulation in anesthetized mice, followed by repeated instillation and aspiration of sterile saline. The collected BALF was centrifuged to remove debris, and the supernatant was stored for further analysis. Cells from the BALF pellet were fixed onto glass slides and stained with a Wright-Giemsa kit according to the manufacturer’s instructions. Leukocyte populations were then identified and evaluated under a light microscope to assess inflammatory cell infiltration in the lung.

### Preparation and characterization of PEVs@PLGA@si-CLYBL nanoparticles

Polymeric nanoparticles were synthesized using a double emulsion-solvent evaporation method with PLGA dissolved in dichloromethane and emulsified with 1% polyvinyl alcohol (PVA). The double emulsion was formed by ultrasonic homogenization and subsequent addition to PVA, followed by solvent evaporation. Nanoparticles were collected by high-speed centrifugation, washed, and resuspended in PBS. For fluorescent labeling, rhodamine was added during synthesis. Particle size and zeta potential were measured by dynamic light scattering (DLS), while morphology was observed by transmission electron microscopy (TEM).

CLYBL siRNA was complexed with polyethyleneimine (PEI) at an N/P ratio of 6:1, incubated to form PEI/si-CLYBL complexes, and then combined with the PLGA nanoparticle suspension. Sonication and ultracentrifugation ensured encapsulation, and unbound siRNA was removed by washing. Encapsulation efficiency was determined by UV-visible spectrophotometry and typically exceeded 90%. The functional integrity of loaded siRNA was confirmed by RT-qPCR.

Platelet-derived extracellular vesicles (PEVs) were isolated from mouse platelet-rich plasma by sequential centrifugation and thrombin activation, followed by ultracentrifugation. For coating, PEVs and PLGA@si-CLYBL nanoparticles were mixed at a 1:10 mass ratio and fused by gentle sonication. The resulting PEVs@PLGA@si-CLYBL nanoparticles were characterized for size, surface charge, and morphology using DLS and TEM, and drug loading efficiency was quantified by fluorescence measurement.

Stability of si-CLYBL and PEVs@PLGA@si-CLYBL was assessed via agarose gel electrophoresis after RNase treatment, and colloidal stability was evaluated by incubating nanoparticles in various physiological media at 37 °C, monitoring particle size changes with DLS to ensure integrity under different conditions.

### Cell culture, experimental grouping, and macrophage isolation

RAW264.7 murine macrophages and MLE-12 lung epithelial cells were cultured in their respective media with 10% FBS and 1% penicillin/streptomycin at 37 °C with 5% CO₂. RAW264.7 cells were grouped as follows: PBS, LPS (1 μg/mL, 24 h), Control (LPS + PBS), PEVs@PLGA@si-CLYBL (LPS + 40 nM PEVs@PLGA@si-CLYBL, 24 h, si-CLYBL: CTGGTAAACAGGTGATCCA (RIBOBIO, China)), PEVs@PLGA@si- SUCLG1 (LPS + 40 nM PEVs@PLGA@si- SUCLG1, 24 h, si-SUCLG1: AGATCTGGCACCCTGACTTAT (RIBOBIO, China)), PEVs@PLGA@si-CLYBL + oe-NC (plus negative control lentivirus), and PEVs@PLGA@si-CLYBL + oe-CLYBL (plus CLYBL-overexpressing lentivirus; AAV-CLYBL, GeneChem, China). For the overexpression construct, several nucleotides at the siRNA target site were mutated using a site-directed mutagenesis kit (QuikChange Lightning, Agilent), generating the sequence CTAGTCAATAGATAATCGA, which encodes the same amino acids but is no longer recognized by si-CLYBL.

Macrophage-epithelial interactions were modeled with a Transwell system (RAW264.7 in the upper, MLE-12 in the lower chamber). Lentiviral transduction was performed using oe-NC and oe-CLYBL (MOI = 10), followed by puromycin selection for stable cell lines [40]. Mouse peritoneal macrophages were isolated by peritoneal lavage, centrifugation, and resuspension in culture medium [41].

### Western blot

Cells or tissue samples were lysed in RIPA buffer containing protease inhibitors (Beyotime, Jiangsu, China). Protein concentrations in the supernatants were determined using the BCA assay. Equal amounts of protein (20–40 µg per lane) were separated by SDS–PAGE and transferred onto PVDF membranes. Membranes were blocked with 5% non-fat milk or 5% BSA for 1 h at room temperature and subsequently incubated with primary antibodies overnight at 4 °C. The primary antibodies used were CLYBL (Thermo Fisher Scientific, Cat# PA5-113267), SUCLG1 (Proteintech, Cat# 14923-1-AP), β-Actin (Invitrogen, Cat# 15G5A11/E2), Cleaved Caspase-3 (Cell Signaling Technology, Cat# 9661), Bax (BD Biosciences, Cat# 556467), and Bcl-2 (Cell Signaling Technology, Cat# 3498). The membranes were washed three times with TBST (10 min each) the following day and incubated with HRP-conjugated secondary antibodies for 1 h at room temperature. The secondary antibodies included HRP-conjugated goat anti-rabbit IgG (H + L) (Thermo Fisher Scientific, Cat# 31460) and HRP-conjugated goat anti-mouse IgG (H + L) (Thermo Fisher Scientific, Cat# 31430). Protein bands were visualized using an ECL detection system, and band intensities were quantified using ImageJ software. β-Actin served as the internal loading control for signal normalization. The Full and uncropped western blots can be found in the supplementary materials.

### Cell function and phenotype assays

Macrophage functions were evaluated by multiple assays. Phagocytosis was assessed using red fluorescent latex bead uptake; RAW264.7 cells were incubated with beads, fixed, stained with Calcein-AM, and analyzed by fluorescence microscopy and ImageJ for quantification. Migration was examined by wound healing: a scratch was made on confluent monolayers, and closure was imaged at 0 and 48 h to calculate migration rates. Chemotactic ability was tested using a Transwell assay, with MCP-1 as chemoattractant in the lower chamber; migrated cells were fixed, stained, and counted in random microscopic fields. For cellular uptake, macrophages were treated with 40 nM rhodamine-labeled PEVs@PLGA@si-CLYBL nanoparticles, and internalization was visualized at various time points under a fluorescence microscope, assessing nanoparticle delivery efficiency.

### Cytotoxicity and biocompatibility assays

Cytotoxicity and biocompatibility of PEVs@PLGA@si-CLYBL nanoparticles were evaluated using multiple methods. RAW264.7 macrophages were exposed to increasing nanoparticle concentrations, and viability was assessed by Calcein-AM/PI double staining and quantified by fluorescence microscopy. Hemolysis assays were performed by incubating nanoparticles with a 5% red blood cell suspension and measuring absorbance to determine hemolysis rates, thus evaluating systemic biosafety. Cell proliferation and viability of both RAW264.7 and MLE-12 cells were measured using the CCK-8 assay, with OD values reflecting cell growth. Cellular membrane integrity was assessed by quantifying LDH release into the culture supernatant using a commercial kit; absorbance readings indicated the extent of cell damage. Collectively, these assays provided a comprehensive evaluation of cytotoxicity and compatibility for in vitro applications.

### RNA sequencing and bioinformatics analysis

Lung tissues from the Sepsis group (CLP-induced) and the Treat group (PEVs@PLGA@si-CLYBL, 0.6 mg/kg, post-CLP, *n* = 3 per group) were collected for transcriptome analysis. Total RNA was extracted with TRIzol reagent, and quality was verified using a NanoDrop 2000 spectrophotometer (A260/A280 = 1.8–2.0). Libraries were constructed and sequenced on the Illumina NovaSeq 6000 platform (20 Gb/sample). Raw FASTQ data underwent quality control with FastQC v0.11.9, and low-quality reads were removed. Clean reads were aligned to the mouse reference genome using HISAT2 (v2.2.1), and differential expression analysis was performed with DESeq2 (v1.26.0). Genes with *p* < 0.05 and log₂FC > 1 were considered significantly differentially expressed (Table S1). Functional enrichment of differentially expressed genes (DEGs) was conducted using ClusterProfiler (v3.14.3), including Gene Ontology (GO) and KEGG pathway analyses to identify relevant biological processes and signaling pathways. Immune cell infiltration in lung tissues was analyzed with the CIBERSORT algorithm to assess the immunomodulatory effects of PEVs@PLGA@si-CLYBL (Table S2).

### Liquid chromatography–mass spectrometry (LC–MS) analysis of metabolite levels

LC–MS was employed to quantify the levels of itaconate or citramalyl-CoA (Table S3) in mouse lung tissues, peritoneal macrophages, and the murine macrophage cell line RAW264.7. The procedure was performed as previously described [42], with minor modifications. Briefly, samples were homogenized in ice-cold extraction buffer (acetonitrile:methanol:H_2_O = 67.5:22.5:10, v/v/v), spiked with an internal standard [^13^C5]-itaconate (Eurisotop, CLM-9966-0.001), and transferred to Eppendorf tubes. The mixtures were vortexed and centrifuged at 13,200 rpm for 10 min at 4 °C. Subsequently, 100 µL of the supernatant was transferred into LC–MS glass vials for analysis.

Metabolites were separated on an Xbridge BEH Amide XP HILIC column (2.5 µm, 2.1 × 100 mm; Waters, 186006091) maintained at 27°C. Mobile phase A consisted of 20 mM ammonium acetate with 0.25% ammonium hydroxide (pH 9.0), and mobile phase B was 100% acetonitrile. The flow rate was set to 220 µL/min, and the gradient elution program was as follows: 0 min, 85% B; 0.5 min, 85% B; 9 min, 35% B; 11 min, 2% B; 12 min, 85% B; and 25 min, 85% B. Mass spectrometry was performed in negative ion mode with a full scan range of 70–1000 m/z. The spray voltage was set at 2.5 kV, the heated capillary temperature at 310 °C, and the HESI probe temperature at 370 °C. The sheath gas, auxiliary gas, and sweep gas flow rates were 50, 10, and 2 units, respectively. The resolution was set to 140,000, with an AGC target of 3 × 10⁶ and a maximum injection time of 400 ms. The sample injection order was randomized. Peak picking, alignment, and integration were conducted using Progenesis QI software (Waters, NC).

### Semi-targeted metabolomics analysis

Semi-targeted metabolomics analysis was performed on LPS-stimulated RAW264.7 murine macrophages (Control group) and LPS-stimulated RAW264.7 cells treated with PEVs@PLGA@si-CLYBL nanoparticles (Treatment group) (Table S4). Each group included six biological replicates (*n* = 6).

Cells were washed with PBS (Thermo Fisher Scientific, USA, Cat#10010023), and cellular metabolism was rapidly quenched in liquid nitrogen. Metabolites were extracted using prechilled 80% methanol (v/v) (Sigma-Aldrich, USA, Cat#34860), followed by ice-bath ultrasonication (Bioruptor UCD-200, Diagenode, Belgium) and centrifugation (15,000 × *g*, 4 °C, 10 min; Eppendorf 5424 R, Germany). The supernatants were dried under nitrogen (Reacti-Vap, Thermo Fisher Scientific, USA), reconstituted in 50% methanol containing 0.1% formic acid, and filtered through 0.22 µm membranes (Millipore, USA, Cat#SLGV013NL) prior to analysis.

Metabolite detection was conducted using a Vanquish UHPLC system (Thermo Fisher Scientific, USA) coupled to a Q Exactive HF-X high-resolution mass spectrometer (Thermo Fisher Scientific, USA), equipped with a Waters ACQUITY UPLC HSS T3 column (2.1 × 100 mm, 1.8 µm, UK). The mobile phases consisted of water containing 0.1% formic acid (A) and acetonitrile containing 0.1% formic acid (B). Mass spectrometry was performed in alternating positive and negative ion modes, with signal normalization carried out using internal standards. Authentic metabolite standards were obtained from Sigma-Aldrich (USA), Cayman Chemical (USA), and Toronto Research Chemicals (Canada) to assist metabolite annotation and signal calibration.

Raw data were processed using Xcalibur 4.3 and TraceFinder 5.1 software for peak extraction and peak area integration. Metabolites were putatively annotated based on accurate mass, retention time, and comparison with the HMDB, KEGG, and mzCloud databases. Features with relative standard deviation (RSD) < 15% in quality control (QC) samples were retained for subsequent analyses. Differential metabolites between groups were identified using two-tailed t-tests with Benjamini-Hochberg multiple testing correction (false discovery rate [FDR] < 0.05, |log₂ fold change|> 1). Metabolic pathway enrichment analysis was performed using MetaboAnalyst 6.0 (https://www.metaboanalyst.ca).

### Reactive oxygen species (ROS) and apoptosis analysis

Intracellular ROS levels were assessed by incubating treated cells with DCFH-DA and quantifying fluorescence intensity under a microscope. Apoptosis was measured using Annexin V-FITC/PI dual staining followed by flow cytometry; cells were stained after treatment, incubated, and analyzed to determine the proportions of early and late apoptotic populations. These assays enabled evaluation of oxidative stress and apoptotic response under different experimental conditions.

### Enzyme-linked immunosorbent assay (ELISA)

To quantify the levels of inflammatory cytokines TNF-α, IL-6, and IL-1β in cell culture supernatants, BALF, and lung tissue homogenates, an ELISA was conducted. After treatment, culture supernatants were collected and centrifuged at 3000 rpm for 10 min at 4 °C to remove debris. The supernatants were transferred to new tubes and stored at –80 °C until analysis. BALF samples were used directly for ELISA. For lung tissue samples, the tissues were rinsed with cold PBS, snap-frozen in liquid nitrogen, and subsequently homogenized in lysis buffer containing protease inhibitors. Homogenates were centrifuged at 12,000 × *g* for 10 min at 4 °C, and the supernatants were collected. Commercial ELISA kits specific for mouse TNF-α (D721150-0048), IL-6 (D721022-0048), and IL-1β (D721017-0048) (Sangon Biotech, China) were used according to the manufacturer’s protocols.

### siRNA release profile

To mimic the in vivo release behavior of siRNA, FAM-labeled PEVs@PLGA@si-CLYBL nanoparticles (40 nM) were incubated in RPMI-1640 medium (Gibco, USA) under different pH conditions—pH 7.4 (physiological) and pH 5.0 (endosomal). At predetermined time points, samples were collected, and the fluorescence intensity of released FAM-siRNA was measured using a fluorescence microplate reader (Tecan, Switzerland). The cumulative release profile was plotted to assess release kinetics and total siRNA release under varying pH environments.

### Statistical analysis

All data were derived from at least three independent experiments and are presented as mean ± standard deviation (SD). For comparisons between two groups, an unpaired Student’s *t* test was used. For comparisons among three or more groups, one-way analysis of variance (ANOVA) was applied. If ANOVA detected significant differences, Tukey’s honest significant difference test was further used for pairwise comparisons. For data not conforming to a normal distribution or homogeneous variance, the Mann-Whitney U test or Kruskal-Wallis H test was employed. All statistical analyses were performed using GraphPad Prism version 9.5.0 (GraphPad Software, Inc.) and R version 4.2.1 (R Foundation for Statistical Computing). A two-tailed *p*-value < 0.05 was considered statistically significant, while *p* ≥ 0.05 was regarded as not significant.

## Supplementary information

Full-length, uncropped original Western blots

Supplemental Materials

## Data Availability

All data generated or analyzed during this study are included in this article and/or its supplementary material files. Further inquiries can be directed to the corresponding author.
