# Supplementary material for: Targeted silencing of CLYBL with platelet-mimetic siRNA nanoparticles drives itaconate–mediated macrophage reprogramming and protects against sepsis-triggered lung cell death
Source: Cell Death Discov. 2026 May 30;12:321. doi: 10.1038/s41420-026-03119-6 (PMC13429697; doi:10.1038/s41420-026-03119-6)
Supplement: Supplementary file 1 — Full-length, uncropped original Western blots [file 41420_2026_3119_MOESM1_ESM.docx]

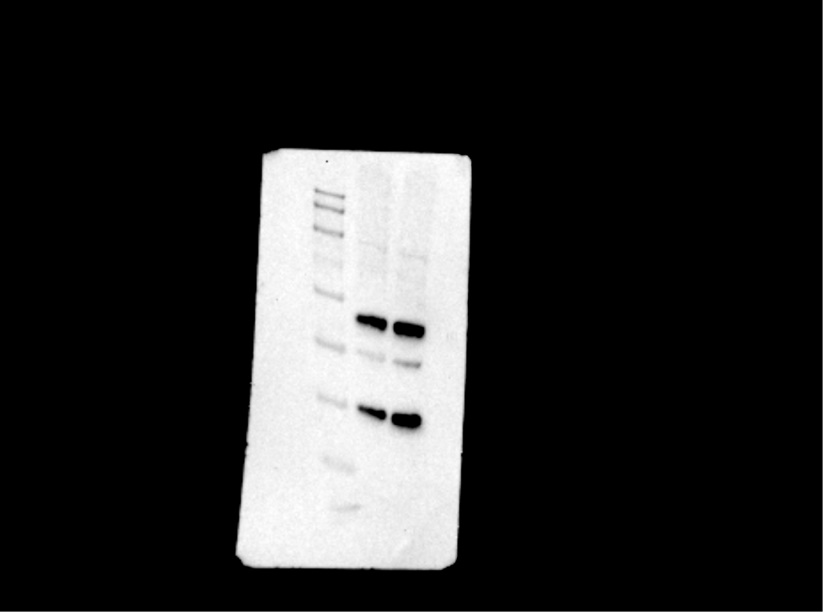


Figure 1C-1


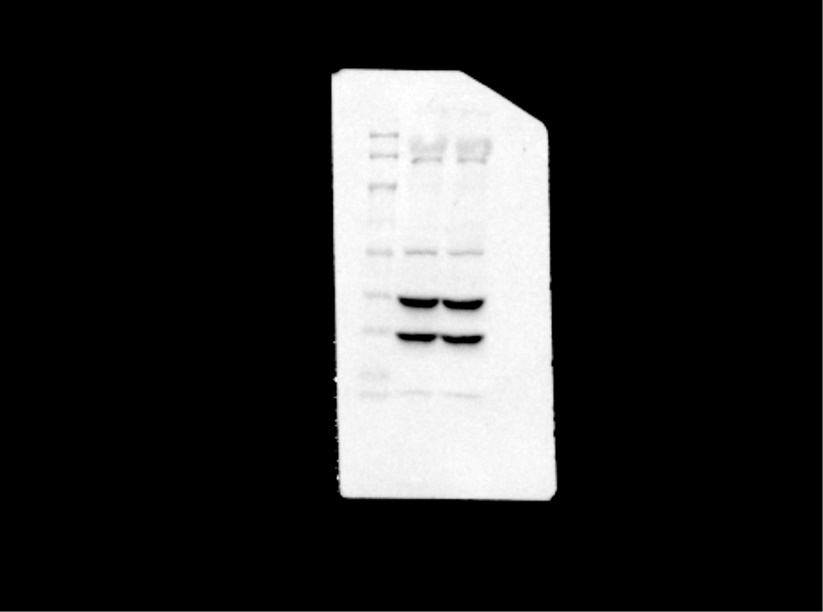


Figure 1C-2


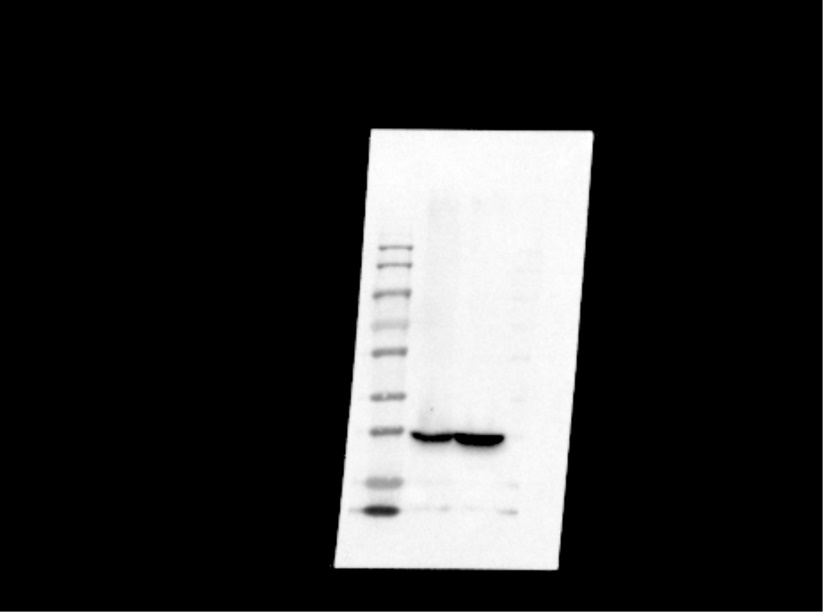


Figure 1G-1


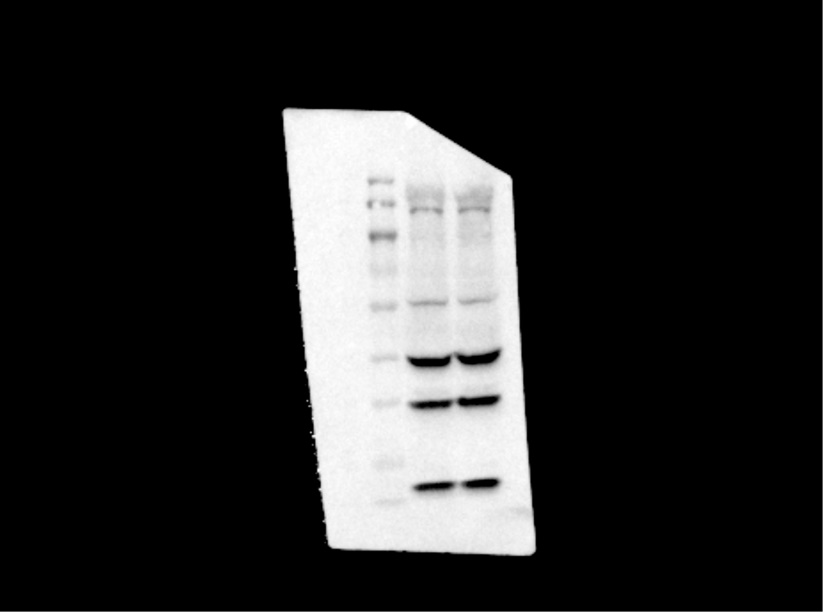


Figure 1G-2


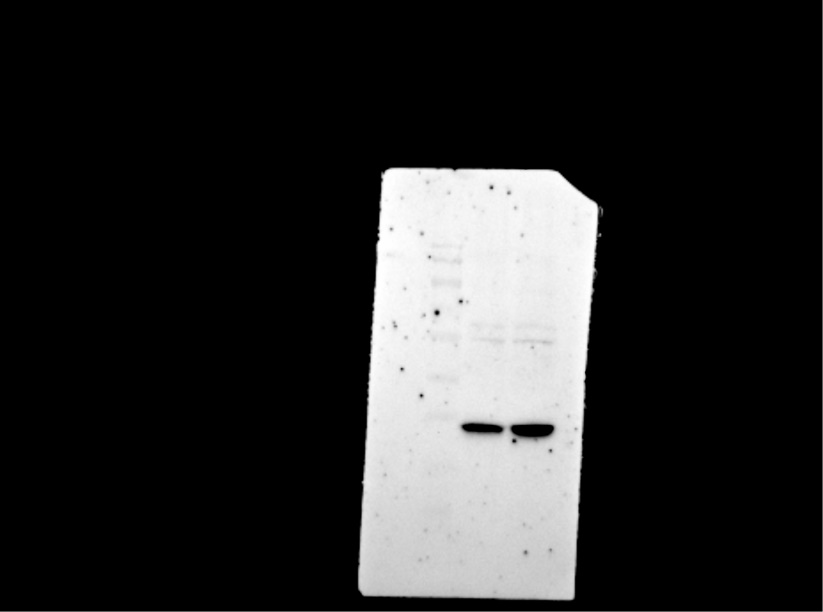


Figure 1K-1


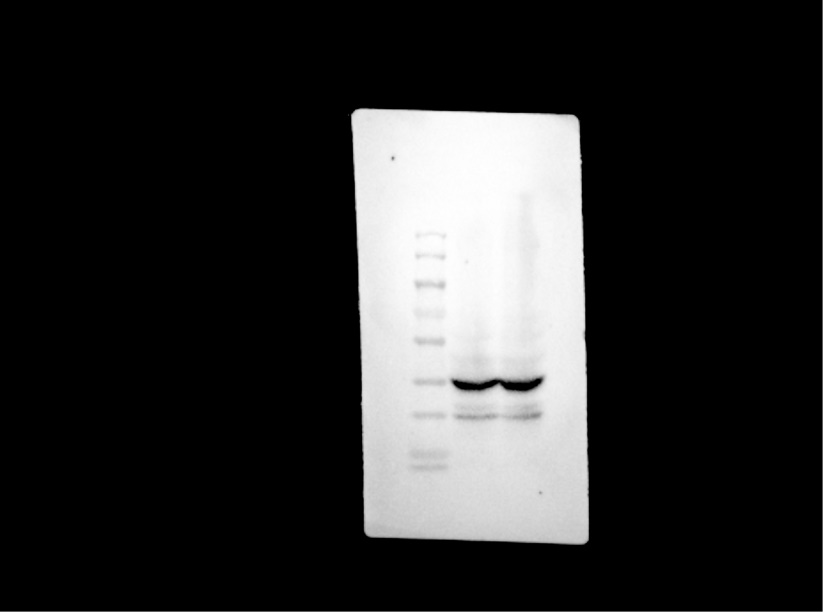


Figure 1K-2


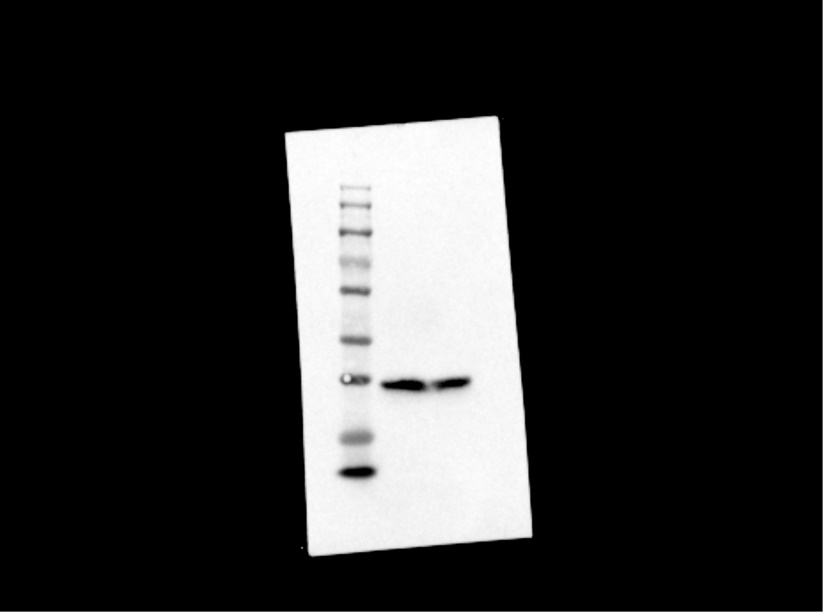


Figure 4C-1


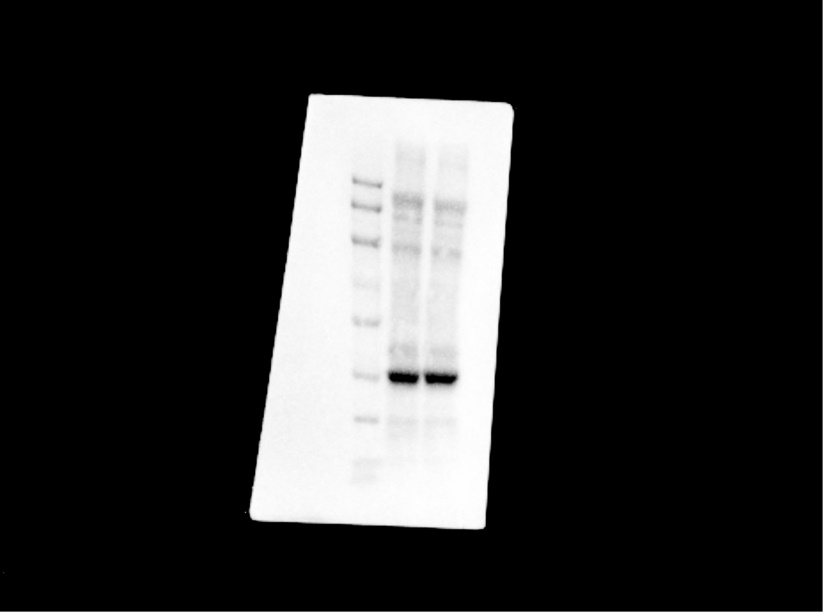


Figure 4C-2


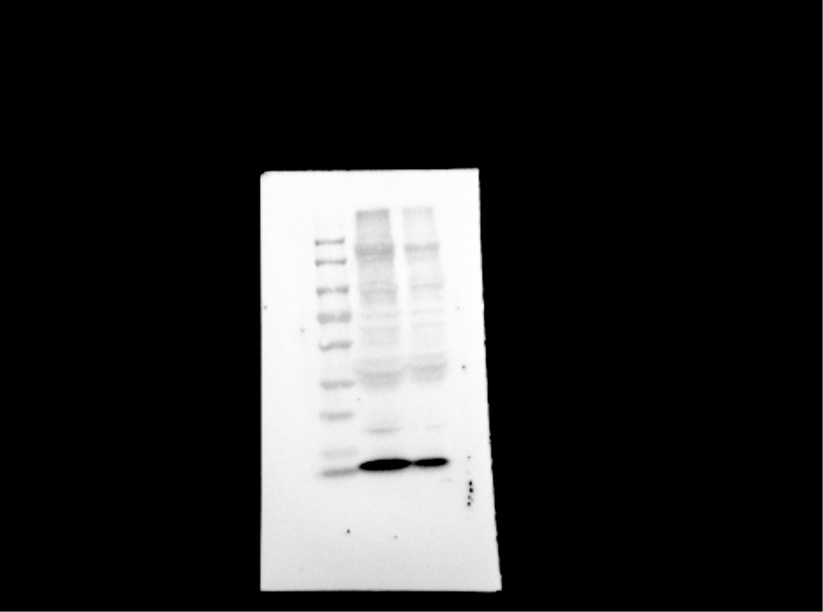


Figure 5D-1


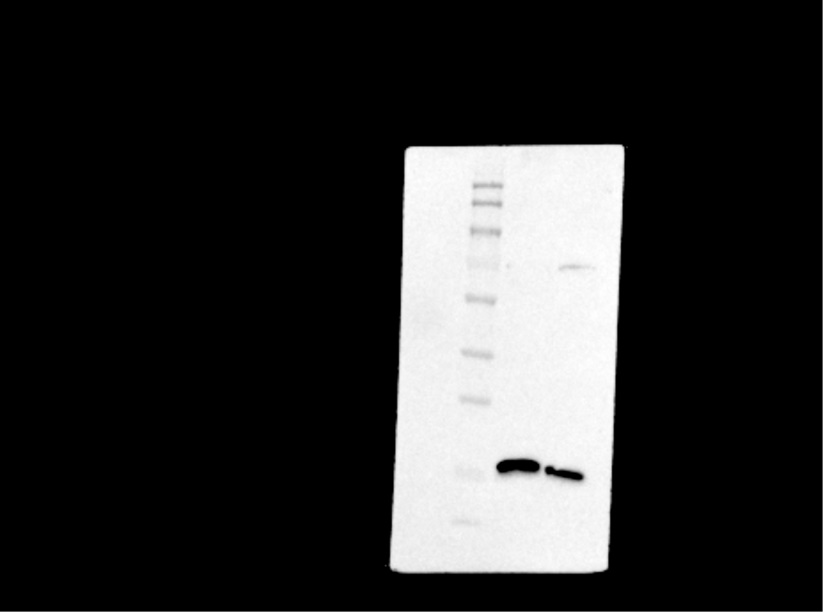


Figure 5D-2


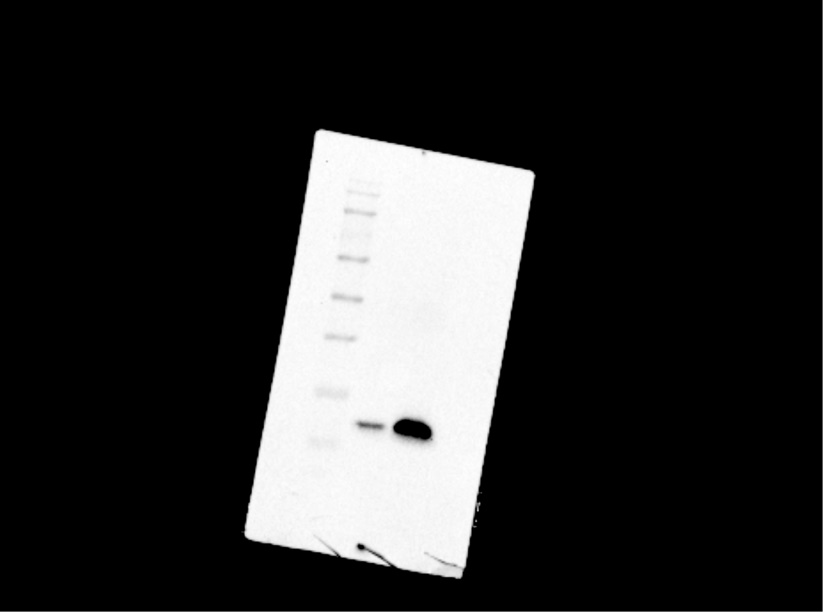


Figure 5D-3


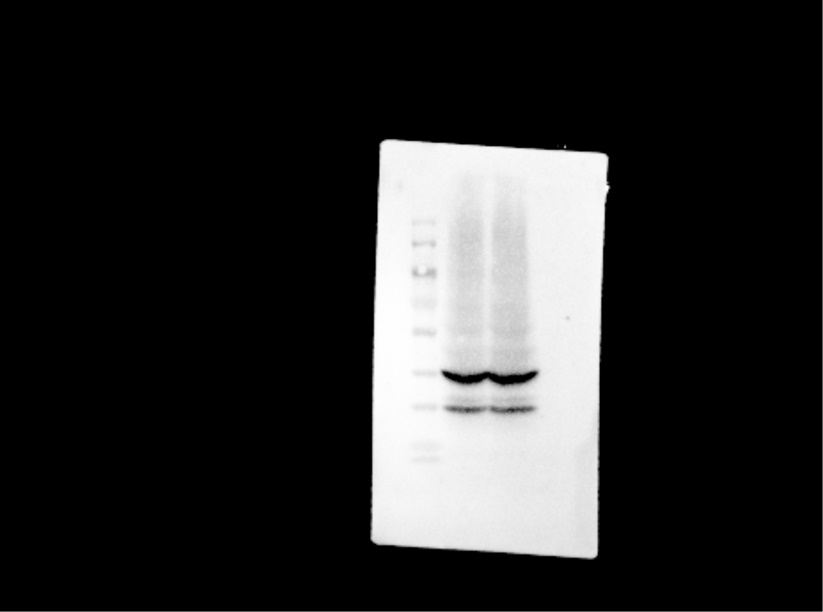


Figure 5D-4


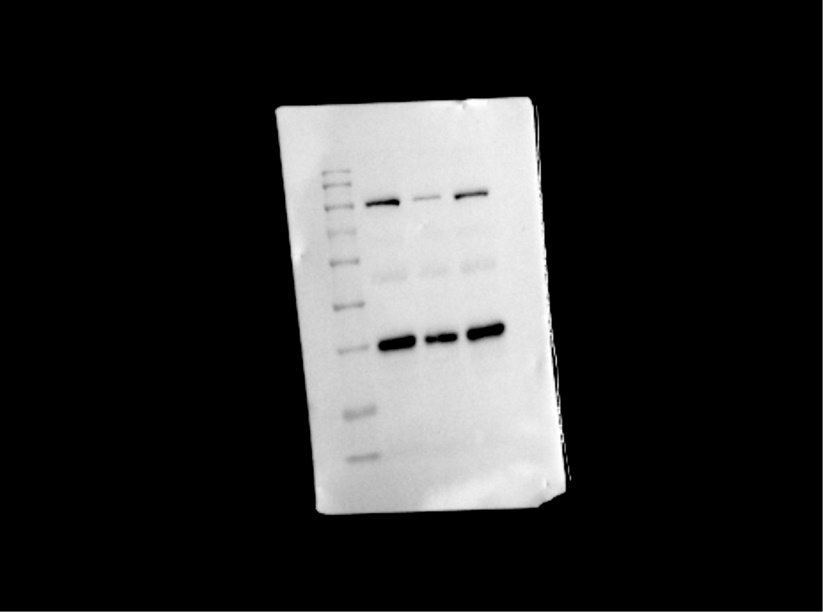


Figure 7H-1


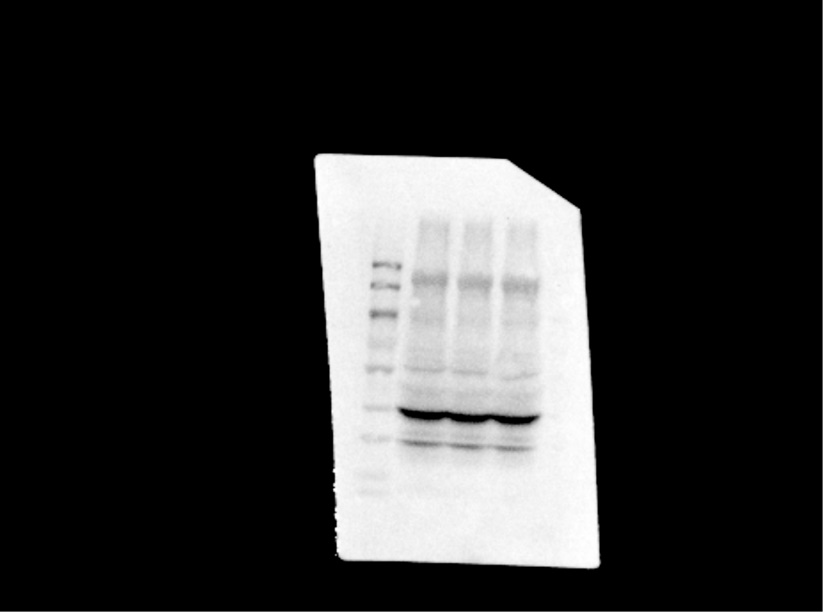


Figure 7H-2


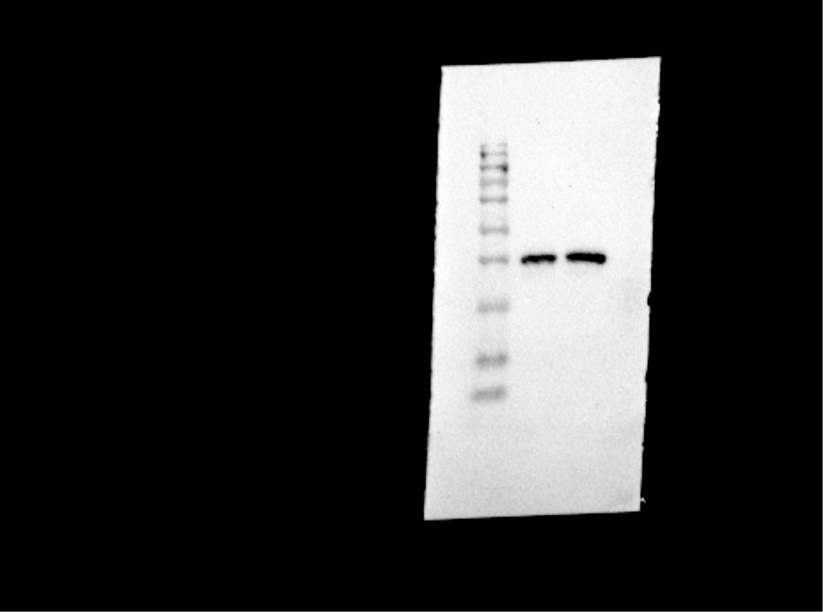


Figure S2B-1


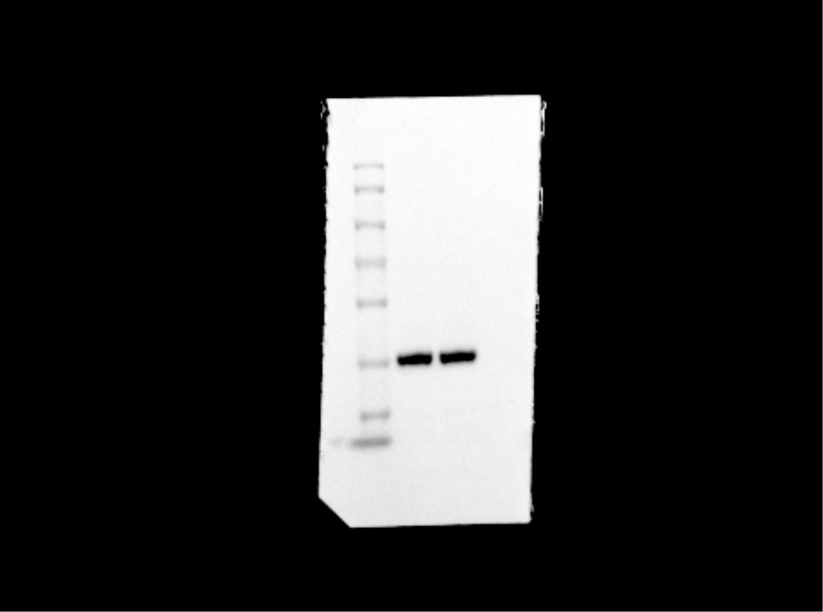


Figure S2B-2


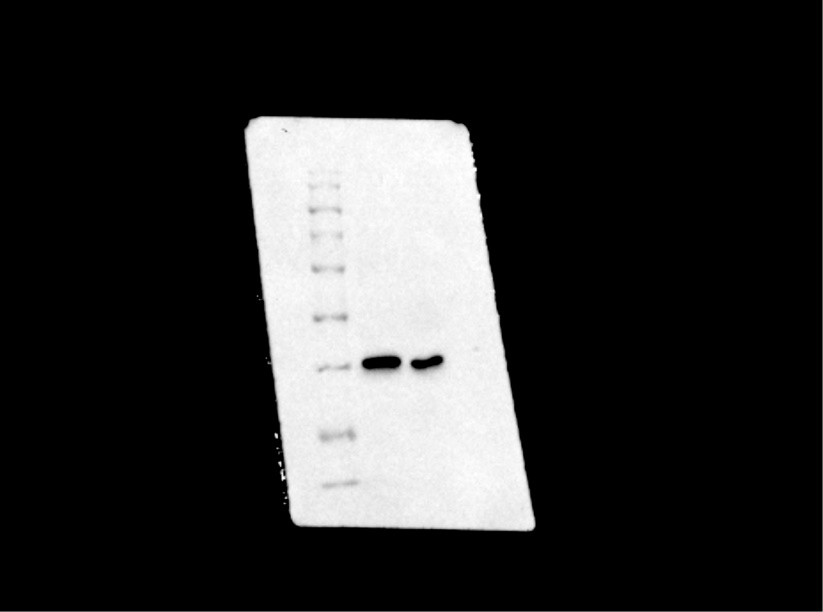


Figure S2D-1


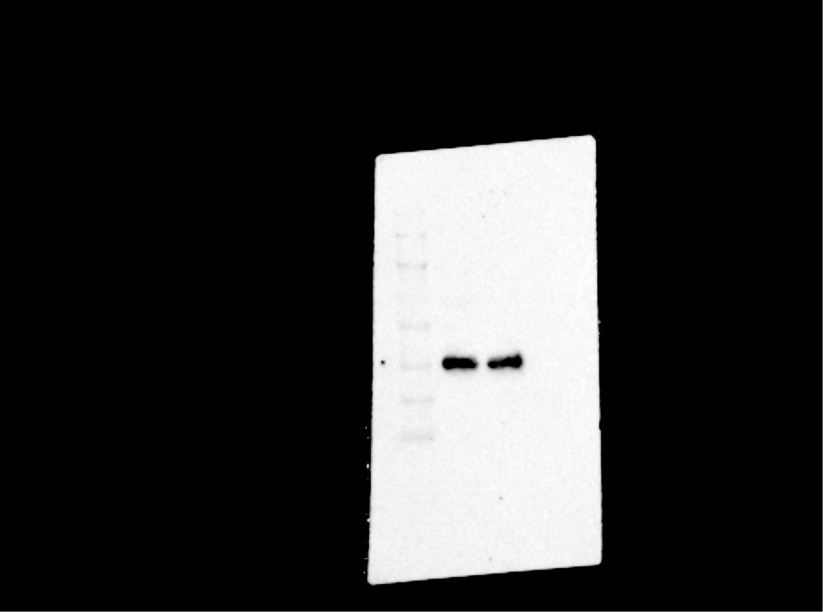


Figure S2D-2


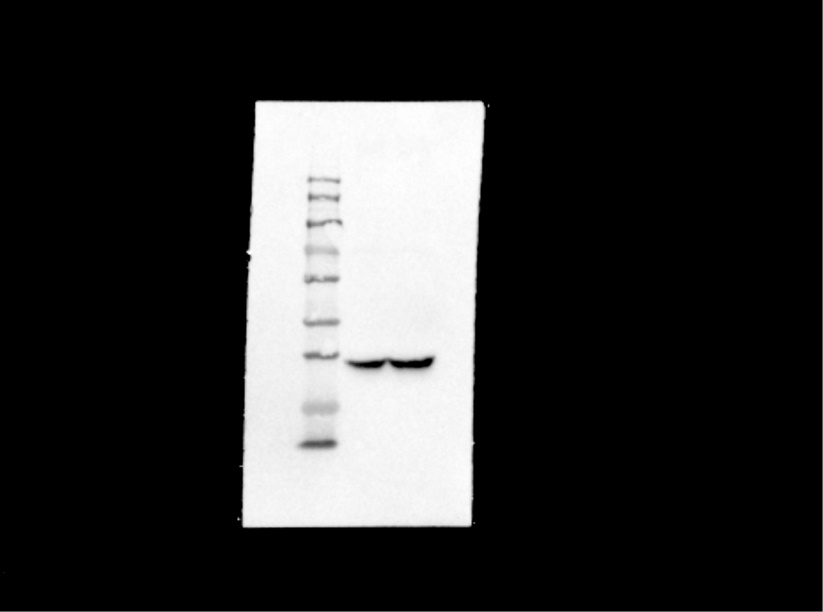


Figure S3B-1


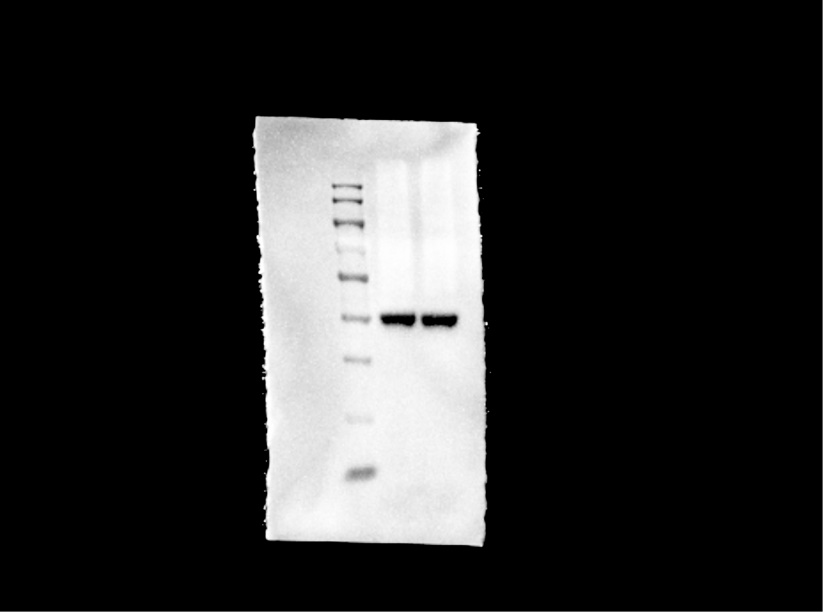


Figure S3B-2


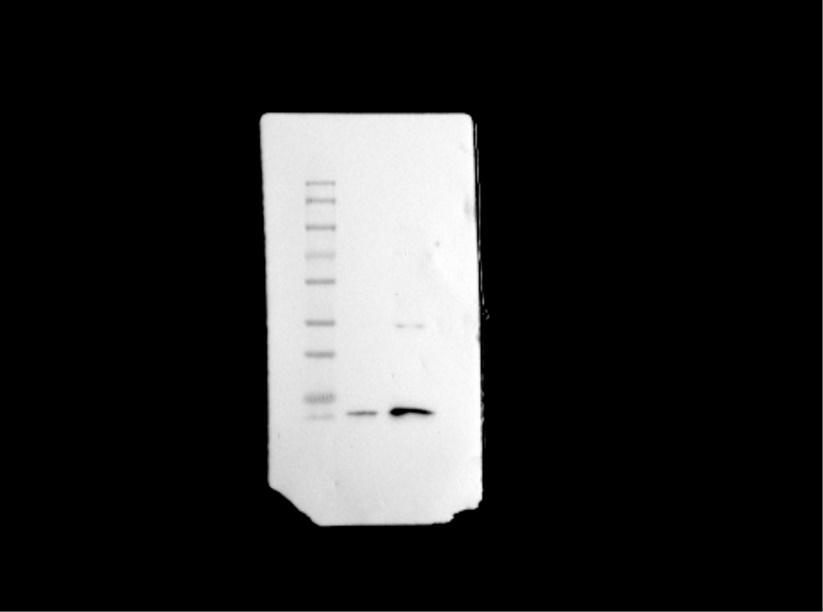


Figure S4C-1


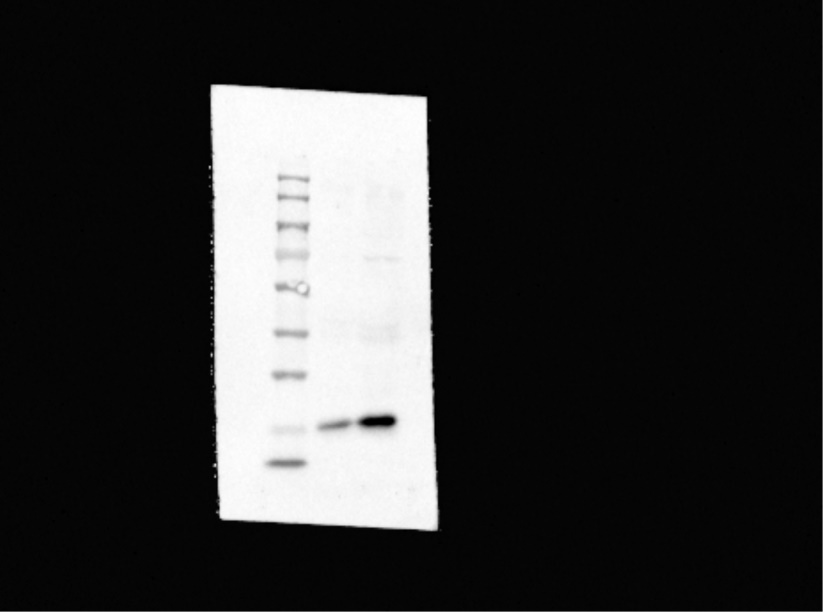


Figure S4C-2


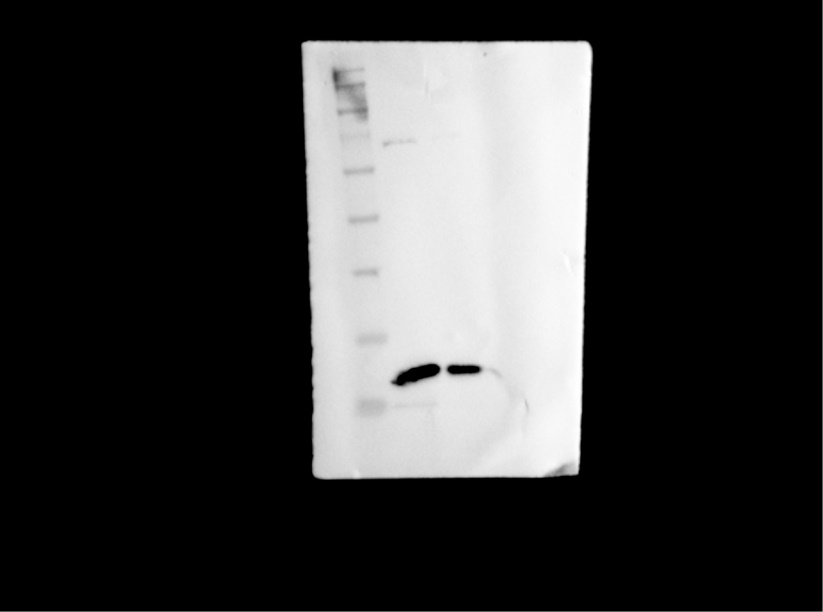


Figure S4C-3


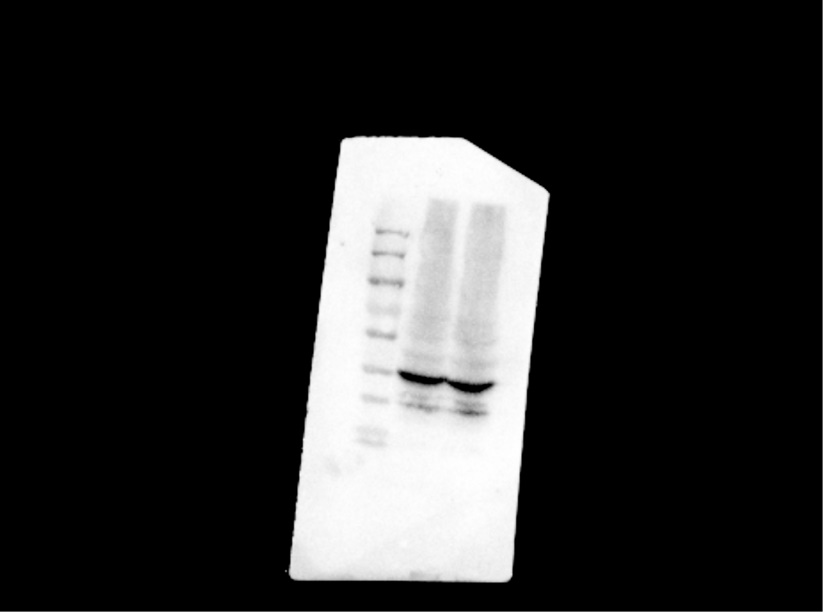


Figure S4C-4


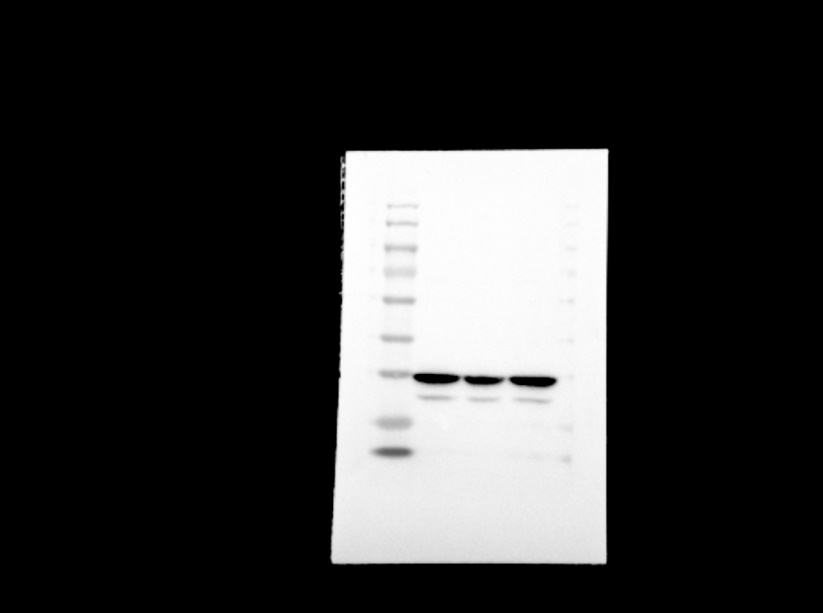


Figure S5A-1


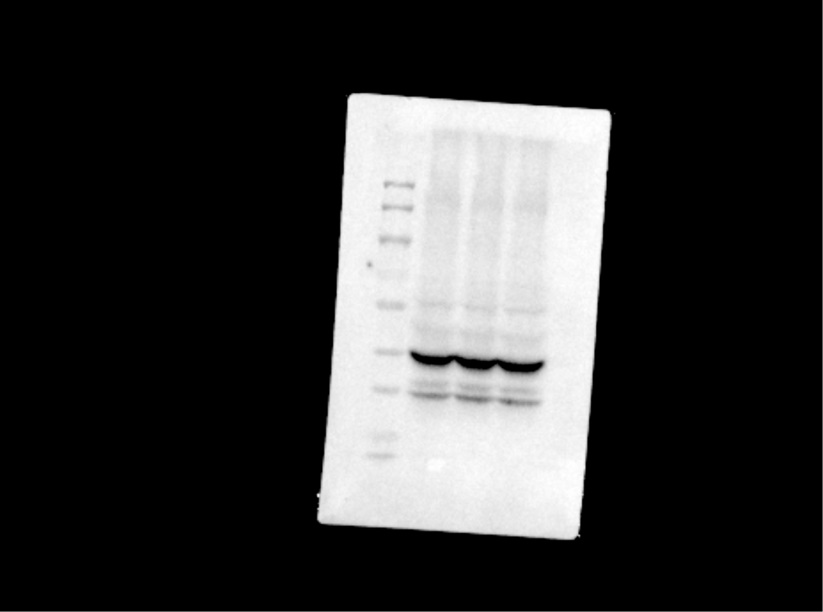


Figure S5A-2
