# Supplementary material for: Targeted silencing of CLYBL with platelet-mimetic siRNA nanoparticles drives itaconate–mediated macrophage reprogramming and protects against sepsis-triggered lung cell death
Source: Cell Death Discov. 2026 May 30;12:321. doi: 10.1038/s41420-026-03119-6 (PMC13429697; doi:10.1038/s41420-026-03119-6)
Supplement: Supplementary file 2 — Supplemental Materials [file 41420_2026_3119_MOESM2_ESM.docx]

**Table S1. Sequence of RT-qPCR.**

| **Name** | **Sequence (5’-3’)** |
| --- | --- |
| CLYBL(mouse) | Forward: GCCATGGGCTTCACTGGTAA |
|  | Reverse: GCCTGAGGAGGGCTTCATTT |
| TNF-α (mouse） | Forward: GTAGCCCACGTCGTAGCAAA |
|  | Reverse: TAGCAAATCGGCTGACGGTG |
| IL-6(mouse） | Forward: CTCTGGAGCCCACCAAGAAC |
|  | Reverse: TGCCATTGCACAACTCTTTTC |
| IL-1β(mouse） | Forward: TGCCACCTTTTGACAGTGATG |
|  | Reverse: AAGGTCCACGGGAAAGACAC |
| β-actin (mouse) | Forward: GTCGAGTCGCGTCCACC |
|  | Reverse: CGATGGAGGGGAATACAGCC |

Note: Abbreviations: F, forward; R, reverse.

**Table S2. Primary antibody information.**

| **Name** | **Manufactor** | **Cat.NO** | **Dilution ratio** |
| --- | --- | --- | --- |
| CLYBL | Abcam | Ab235434 | 1:5000 |
| cleaved-caspase-3 | Cell Signaling Technology, | 9661T | 1:1000 |
| Bcl2 | Cell Signaling Technology, | 3498 | 1:1000 |
| Bax | Cell Signaling Technology, | 2772 | 1:1000 |
| β-actin | Abcam | ab8226 | 1:10000 |

**Table S3. Targeted LC–MS information table**

| **Metabolite** | **LC–MS method** | **Chromatography** | **Ion mode** | **RT (min)** | **Precursor m/z** | **MS2 fragments** | **Quantifier / Qualifier ion** | **Identification confidence** |
| --- | --- | --- | --- | --- | --- | --- | --- | --- |
| Itaconate | Targeted LC–MS | HILIC (XBridge BEH Amide XP) | Negative | ~2–3* | 129.018 ([M–H]⁻) | Characteristic fragments consistent with reference spectra | 129.018 (MS1), quantified using [¹³C₅]-itaconate internal standard | **Level 1** |
| Citramalyl-CoA | Targeted LC–MS | HILIC (XBridge BEH Amide XP) | Negative | ~7–9* | ~884 ([M–H]⁻)** | Not systematically acquired / not used for quantification | MS1 extracted ion | **Level 2** |

**Table S4. Semi-targeted LC–MS information table**

| **Metabolite** | **LC–MS method** | **Chromatography** | **Ion mode** | **RT (min)** | **Precursor m/z** | **MS2 fragments** | **Identification confidence** |
| --- | --- | --- | --- | --- | --- | --- | --- |
| Alanine | Semi-targeted LC–MS | RP-LC (Waters ACQUITY UPLC HSS T3) | Positive/Negative (polarity switching) | 1.2 | 88.0393 | 44, 60 | Level 2 |
| Glutamine | Semi-targeted LC–MS | RP-LC (Waters ACQUITY UPLC HSS T3) | Positive/Negative (polarity switching) | 2.4 | 147.0764 | 84, 130 | Level 2 |
| Proline | Semi-targeted LC–MS | RP-LC (Waters ACQUITY UPLC HSS T3) | Positive/Negative (polarity switching) | 1.5 | 116.0706 | 70, 98 | Level 2 |
| Threonine | Semi-targeted LC–MS | RP-LC (Waters ACQUITY UPLC HSS T3) | Positive/Negative (polarity switching) | 1.7 | 120.0655 | 74, 102 | Level 2 |
| Glycine | Semi-targeted LC–MS | RP-LC (Waters ACQUITY UPLC HSS T3) | Positive/Negative (polarity switching) | 0.9 | 76.0393 | 30, 48 | Level 2 |
| Methionine | Semi-targeted LC–MS | RP-LC (Waters ACQUITY UPLC HSS T3) | Positive/Negative (polarity switching) | 2.3 | 150.058 | 104, 132 | Level 2 |
| Serine | Semi-targeted LC–MS | RP-LC (Waters ACQUITY UPLC HSS T3) | Positive/Negative (polarity switching) | 1 | 106.05 | 60, 88 | Level 2 |
| Aspartate | Semi-targeted LC–MS | RP-LC (Waters ACQUITY UPLC HSS T3) | Positive/Negative (polarity switching) | 1.3 | 132.0302 | 74, 115 | Level 2 |
| Glutamate | Semi-targeted LC–MS | RP-LC (Waters ACQUITY UPLC HSS T3) | Positive/Negative (polarity switching) | 2.5 | 148.06 | 84, 102 | Level 2 |
| Arginine | Semi-targeted LC–MS | RP-LC (Waters ACQUITY UPLC HSS T3) | Positive/Negative (polarity switching) | 2.8 | 175.119 | 70, 130 | Level 2 |
| Citrulline | Semi-targeted LC–MS | RP-LC (Waters ACQUITY UPLC HSS T3) | Positive/Negative (polarity switching) | 2.6 | 176.103 | 70, 113 | Level 2 |
| Nitrate | Semi-targeted LC–MS | RP-LC (Waters ACQUITY UPLC HSS T3) | Positive/Negative (polarity switching) | 0.5 | 62.996 | 46 | Level 2 |
| Nitrite | Semi-targeted LC–MS | RP-LC (Waters ACQUITY UPLC HSS T3) | Positive/Negative (polarity switching) | 0.4 | 46.005 | 30 | Level 2 |
| Ornithine | Semi-targeted LC–MS | RP-LC (Waters ACQUITY UPLC HSS T3) | Positive/Negative (polarity switching) | 2 | 133.097 | 70, 116 | Level 2 |
| 2-Methylbutyrylcarnitine (C5) | Semi-targeted LC–MS | RP-LC (Waters ACQUITY UPLC HSS T3) | Positive/Negative (polarity switching) | 2.7 | 232.1544 | 85, 159 | Level 2 |
| 3-Hydroxyisovalerylcarnitine | Semi-targeted LC–MS | RP-LC (Waters ACQUITY UPLC HSS T3) | Positive/Negative (polarity switching) | 2.6 | 248.149 | 85, 159 | Level 2 |
| Isovalerylcarnitine (C5) | Semi-targeted LC–MS | RP-LC (Waters ACQUITY UPLC HSS T3) | Positive/Negative (polarity switching) | 2.8 | 232.1544 | 85, 159 | Level 2 |
| Tiglylcarnitine (C5:1) | Semi-targeted LC–MS | RP-LC (Waters ACQUITY UPLC HSS T3) | Positive/Negative (polarity switching) | 2.8 | 230.139 | 85, 159 | Level 2 |
| Acetylcarnitine (C2) | Semi-targeted LC–MS | RP-LC (Waters ACQUITY UPLC HSS T3) | Positive/Negative (polarity switching) | 2 | 204.123 | 85, 143 | Level 2 |
| Butyrylcarnitine (C4) | Semi-targeted LC–MS | RP-LC (Waters ACQUITY UPLC HSS T3) | Positive/Negative (polarity switching) | 2.4 | 218.138 | 85, 159 | Level 2 |
| Carnitine (total) | Semi-targeted LC–MS | RP-LC (Waters ACQUITY UPLC HSS T3) | Positive/Negative (polarity switching) | 1.8 | 162.111 | 103, 85 | Level 2 |
| Decanoylcarnitine (C10) | Semi-targeted LC–MS | RP-LC (Waters ACQUITY UPLC HSS T3) | Positive/Negative (polarity switching) | 4.8 | 288.232 | 85, 229 | Level 2 |
| Dodecanoylcarnitine (C12) | Semi-targeted LC–MS | RP-LC (Waters ACQUITY UPLC HSS T3) | Positive/Negative (polarity switching) | 5.5 | 316.263 | 85, 257 | Level 2 |
| Hexanoylcarnitine (C6) | Semi-targeted LC–MS | RP-LC (Waters ACQUITY UPLC HSS T3) | Positive/Negative (polarity switching) | 3.2 | 246.169 | 85, 187 | Level 2 |
| Isobutyrylcarnitine (C4) | Semi-targeted LC–MS | RP-LC (Waters ACQUITY UPLC HSS T3) | Positive/Negative (polarity switching) | 2.4 | 218.138 | 85, 159 | Level 2 |
| Octanoylcarnitine (C8) | Semi-targeted LC–MS | RP-LC (Waters ACQUITY UPLC HSS T3) | Positive/Negative (polarity switching) | 4.2 | 274.201 | 85, 215 | Level 2 |
| ADP | Semi-targeted LC–MS | RP-LC (Waters ACQUITY UPLC HSS T3) | Positive/Negative (polarity switching) | 1.6 | 428.037 | 136, 348 | Level 2 |
| AMP | Semi-targeted LC–MS | RP-LC (Waters ACQUITY UPLC HSS T3) | Positive/Negative (polarity switching) | 1.5 | 348.07 | 136, 250 | Level 2 |
| ATP | Semi-targeted LC–MS | RP-LC (Waters ACQUITY UPLC HSS T3) | Positive/Negative (polarity switching) | 1.7 | 506.995 | 136, 426 | Level 2 |
| 2-Phosphoglycerate | Semi-targeted LC–MS | RP-LC (Waters ACQUITY UPLC HSS T3) | Positive/Negative (polarity switching) | 1.2 | 185.008 | 97, 139 | Level 2 |
| 3-Phosphoglycerate | Semi-targeted LC–MS | RP-LC (Waters ACQUITY UPLC HSS T3) | Positive/Negative (polarity switching) | 1.2 | 185.008 | 97, 139 | Level 2 |
| Dihydroxyacetone phosphate (DHAP) | Semi-targeted LC–MS | RP-LC (Waters ACQUITY UPLC HSS T3) | Positive/Negative (polarity switching) | 0.9 | 169.99 | 97, 123 | Level 2 |
| Fructose-1,6-bisphosphate | Semi-targeted LC–MS | RP-LC (Waters ACQUITY UPLC HSS T3) | Positive/Negative (polarity switching) | 0.9 | 339.9889 | 97, 259 | Level 2 |
| Lactate | Semi-targeted LC–MS | RP-LC (Waters ACQUITY UPLC HSS T3) | Positive/Negative (polarity switching) | 0.8 | 89.0244 | 43, 71 | Level 2 |
| Phosphoenolpyruvate | Semi-targeted LC–MS | RP-LC (Waters ACQUITY UPLC HSS T3) | Positive/Negative (polarity switching) | 1 | 167.9824 | 79, 97 | Level 2 |
| Pyruvate | Semi-targeted LC–MS | RP-LC (Waters ACQUITY UPLC HSS T3) | Positive/Negative (polarity switching) | 0.6 | 87.0088 | 43, 59 | Level 2 |
| Fructose-6-phosphate | Semi-targeted LC–MS | RP-LC (Waters ACQUITY UPLC HSS T3) | Positive/Negative (polarity switching) | 1.3 | 259.022 | 97, 139 | Level 2 |
| Glucose-6-phosphate | Semi-targeted LC–MS | RP-LC (Waters ACQUITY UPLC HSS T3) | Positive/Negative (polarity switching) | 1.3 | 259.022 | 97, 139 | Level 2 |
| Glycerol-3-phosphate | Semi-targeted LC–MS | RP-LC (Waters ACQUITY UPLC HSS T3) | Positive/Negative (polarity switching) | 1 | 171.004 | 97, 123 | Level 2 |
| (S)-Citramalyl-CoA | Semi-targeted LC–MS | RP-LC (Waters ACQUITY UPLC HSS T3) | Positive/Negative (polarity switching) | 7.3 | 900.1 | 428, 261 | Level 3 |
| Itaconate | Semi-targeted LC–MS | RP-LC (Waters ACQUITY UPLC HSS T3) | Positive/Negative (polarity switching) | 3.2 | 129.018 | 85, 101 | Level 2 |
| Itaconyl-CoA | Semi-targeted LC–MS | RP-LC (Waters ACQUITY UPLC HSS T3) | Positive/Negative (polarity switching) | 7.2 | 884.1 | 428, 261 | Level 3 |
| Mesaconate | Semi-targeted LC–MS | RP-LC (Waters ACQUITY UPLC HSS T3) | Positive/Negative (polarity switching) | 3.1 | 129.018 | 85, 101 | Level 3 |
| Cis-aconityl-CoA | Semi-targeted LC–MS | RP-LC (Waters ACQUITY UPLC HSS T3) | Positive/Negative (polarity switching) | 7.1 | 882.1 | 428, 261 | Level 3 |
| cis-Aconitate | Semi-targeted LC–MS | RP-LC (Waters ACQUITY UPLC HSS T3) | Positive/Negative (polarity switching) | 3.4 | 173.009 | 111, 129 | Level 2 |
| Acetyl-CoA | Semi-targeted LC–MS | RP-LC (Waters ACQUITY UPLC HSS T3) | Positive/Negative (polarity switching) | 6.6 | 810.1 | 428, 261 | Level 3 |
| Malonyl-CoA | Semi-targeted LC–MS | RP-LC (Waters ACQUITY UPLC HSS T3) | Positive/Negative (polarity switching) | 6.9 | 854.1 | 428, 261 | Level 3 |
| Formate | Semi-targeted LC–MS | RP-LC (Waters ACQUITY UPLC HSS T3) | Positive/Negative (polarity switching) | 0.5 | 45.992 | 29 | Level 2 |
| Homocysteine | Semi-targeted LC–MS | RP-LC (Waters ACQUITY UPLC HSS T3) | Positive/Negative (polarity switching) | 2.3 | 135.03 | 88, 118 | Level 2 |
| 6-Phosphogluconate | Semi-targeted LC–MS | RP-LC (Waters ACQUITY UPLC HSS T3) | Positive/Negative (polarity switching) | 1.5 | 275.018 | 97, 139 | Level 2 |
| Erythrose-4-phosphate | Semi-targeted LC–MS | RP-LC (Waters ACQUITY UPLC HSS T3) | Positive/Negative (polarity switching) | 1.3 | 199.998 | 97, 139 | Level 2 |
| Ribose-5-phosphate | Semi-targeted LC–MS | RP-LC (Waters ACQUITY UPLC HSS T3) | Positive/Negative (polarity switching) | 1.4 | 229.008 | 97, 139 | Level 2 |
| Sedoheptulose-7-phosphate | Semi-targeted LC–MS | RP-LC (Waters ACQUITY UPLC HSS T3) | Positive/Negative (polarity switching) | 1.6 | 289.029 | 97, 139 | Level 2 |
| Xylulose-5-phosphate | Semi-targeted LC–MS | RP-LC (Waters ACQUITY UPLC HSS T3) | Positive/Negative (polarity switching) | 1.4 | 229.008 | 97, 139 | Level 2 |
| Putrescine | Semi-targeted LC–MS | RP-LC (Waters ACQUITY UPLC HSS T3) | Positive/Negative (polarity switching) | 1.1 | 89.107 | 72, 58 | Level 2 |
| Spermidine | Semi-targeted LC–MS | RP-LC (Waters ACQUITY UPLC HSS T3) | Positive/Negative (polarity switching) | 1.8 | 146.165 | 129, 112 | Level 2 |
| Spermine | Semi-targeted LC–MS | RP-LC (Waters ACQUITY UPLC HSS T3) | Positive/Negative (polarity switching) | 2.2 | 203.222 | 186, 129 | Level 2 |
| 2-Methylcitrate | Semi-targeted LC–MS | RP-LC (Waters ACQUITY UPLC HSS T3) | Positive/Negative (polarity switching) | 3.5 | 191.0197 | 111, 129 | Level 2 |
| C4-DC-carnitine | Semi-targeted LC–MS | RP-LC (Waters ACQUITY UPLC HSS T3) | Positive/Negative (polarity switching) | 2.6 | 248.1182 | 85, 203 | Level 2 |
| Methylmalonyl-CoA | Semi-targeted LC–MS | RP-LC (Waters ACQUITY UPLC HSS T3) | Positive/Negative (polarity switching) | 7.5 | 888.1 | 428, 261 | Level 3 |
| Propionate | Semi-targeted LC–MS | RP-LC (Waters ACQUITY UPLC HSS T3) | Positive/Negative (polarity switching) | 1 | 73.029 | 45, 57 | Level 2 |
| Propionyl-CoA | Semi-targeted LC–MS | RP-LC (Waters ACQUITY UPLC HSS T3) | Positive/Negative (polarity switching) | 7 | 838.1 | 428, 261, 136 | Level 3 |
| Propionyl-carnitine (C3) | Semi-targeted LC–MS | RP-LC (Waters ACQUITY UPLC HSS T3) | Positive/Negative (polarity switching) | 2.2 | 204.1232 | 85, 159 | Level 2 |
| NAD+ | Semi-targeted LC–MS | RP-LC (Waters ACQUITY UPLC HSS T3) | Positive/Negative (polarity switching) | 3.4 | 662.1 | 540, 136 | Level 2 |
| NADH | Semi-targeted LC–MS | RP-LC (Waters ACQUITY UPLC HSS T3) | Positive/Negative (polarity switching) | 3.6 | 664.1 | 542, 136 | Level 2 |
| NADP+ | Semi-targeted LC–MS | RP-LC (Waters ACQUITY UPLC HSS T3) | Positive/Negative (polarity switching) | 3.6 | 744.1 | 428, 136 | Level 2 |
| NADPH | Semi-targeted LC–MS | RP-LC (Waters ACQUITY UPLC HSS T3) | Positive/Negative (polarity switching) | 3.7 | 746.1 | 428, 136 | Level 2 |
| Cysteine | Semi-targeted LC–MS | RP-LC (Waters ACQUITY UPLC HSS T3) | Positive/Negative (polarity switching) | 1.9 | 122.027 | 76, 104 | Level 2 |
| GSH (reduced) | Semi-targeted LC–MS | RP-LC (Waters ACQUITY UPLC HSS T3) | Positive/Negative (polarity switching) | 3.1 | 306.0766 | 179, 162 | Level 1 |
| GSSG (oxidized) | Semi-targeted LC–MS | RP-LC (Waters ACQUITY UPLC HSS T3) | Positive/Negative (polarity switching) | 4 | 613.2 | 306, 484 | Level 2 |
| Ophthalmate | Semi-targeted LC–MS | RP-LC (Waters ACQUITY UPLC HSS T3) | Positive/Negative (polarity switching) | 2.6 | 290.09 | 143, 162 | Level 2 |
| γ-Glutamylcysteine | Semi-targeted LC–MS | RP-LC (Waters ACQUITY UPLC HSS T3) | Positive/Negative (polarity switching) | 2.8 | 249.058 | 130, 162 | Level 2 |
| GSH–itaconate adduct | Semi-targeted LC–MS | RP-LC (Waters ACQUITY UPLC HSS T3) | Positive/Negative (polarity switching) | 3.6 | 411.1 | 179, 306 | Level 3 |
| Citrate | Semi-targeted LC–MS | RP-LC (Waters ACQUITY UPLC HSS T3) | Positive/Negative (polarity switching) | 3.6 | 191.0197 | 111, 129 | Level 2 |
| Fumarate | Semi-targeted LC–MS | RP-LC (Waters ACQUITY UPLC HSS T3) | Positive/Negative (polarity switching) | 2.9 | 115.0031 | 71, 87 | Level 2 |
| Isocitrate | Semi-targeted LC–MS | RP-LC (Waters ACQUITY UPLC HSS T3) | Positive/Negative (polarity switching) | 3.5 | 191.0197 | 111, 129 | Level 2 |
| Malate | Semi-targeted LC–MS | RP-LC (Waters ACQUITY UPLC HSS T3) | Positive/Negative (polarity switching) | 3.2 | 133.0136 | 115, 71 | Level 2 |
| Oxaloacetate | Semi-targeted LC–MS | RP-LC (Waters ACQUITY UPLC HSS T3) | Positive/Negative (polarity switching) | 2.6 | 131.0092 | 87, 115 | Level 2 |
| Succinate | Semi-targeted LC–MS | RP-LC (Waters ACQUITY UPLC HSS T3) | Positive/Negative (polarity switching) | 3 | 117.018 | 73, 99 | Level 2 |
| α-Ketoglutarate | Semi-targeted LC–MS | RP-LC (Waters ACQUITY UPLC HSS T3) | Positive/Negative (polarity switching) | 2.9 | 145.014 | 101, 115 | Level 2 |
| Succinyl-CoA | Semi-targeted LC–MS | RP-LC (Waters ACQUITY UPLC HSS T3) | Positive/Negative (polarity switching) | 6.8 | 860.1 | 428, 136 | Level 3 |

**
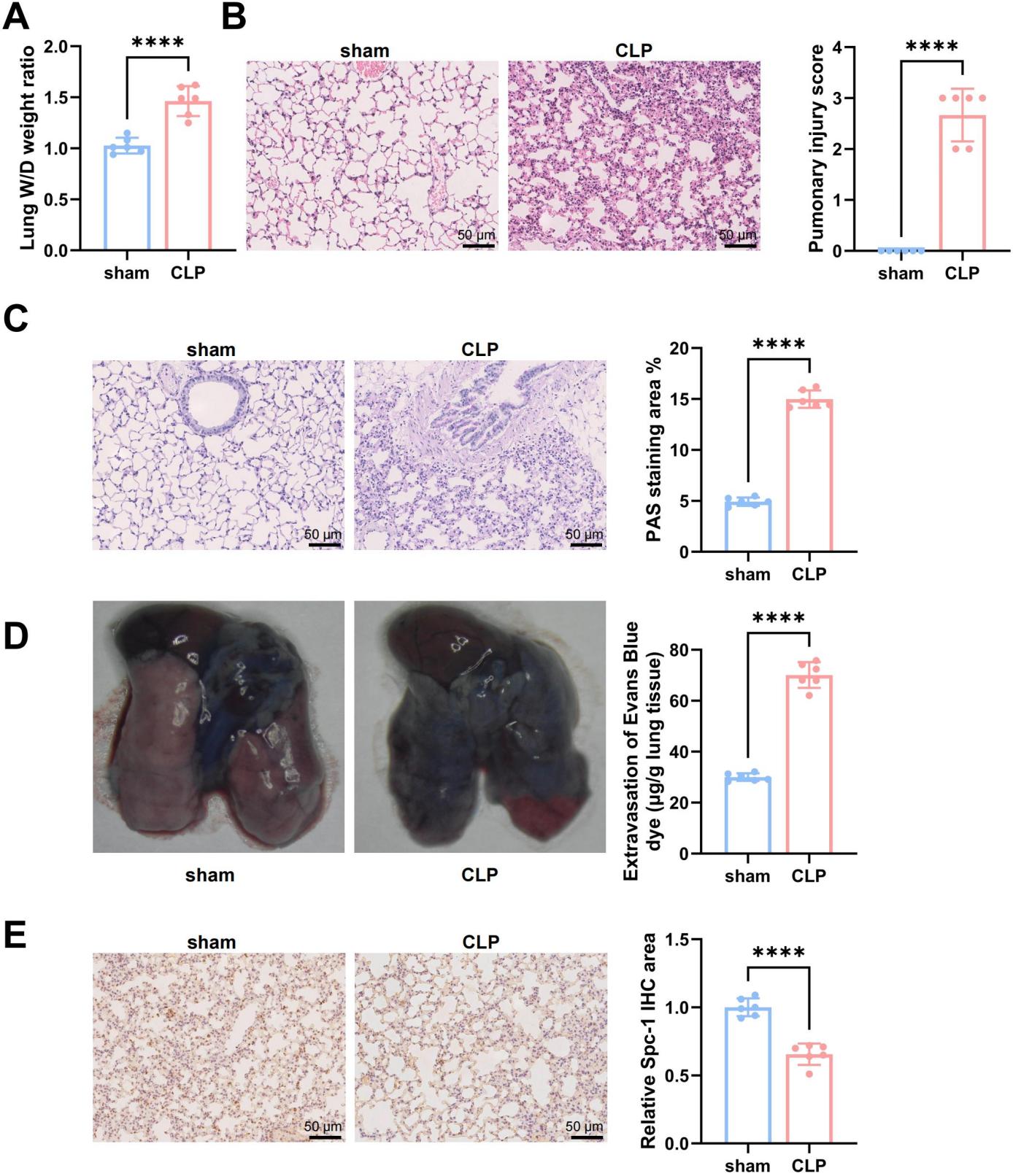
**

**Figure S1. Establishment and pathological evaluation of the sepsis-induced lung injury model.**

Note: (A) Assessment of pulmonary edema by measuring the lung W/D weight ratio in CLP and sham groups; (B) H&E staining to evaluate histopathological changes in lung tissues (scale bar: 50 μm); (C) PAS staining to observe epithelial cell shedding (scale bar: 50 μm); (D) Evans blue staining to assess vascular permeability in lung tissues; (E) IHC analysis of Spc-1 expression intensity (scale bar: 50 μm). All experiments were repeated three times with six animals per group. *****p*<0.0001.

**
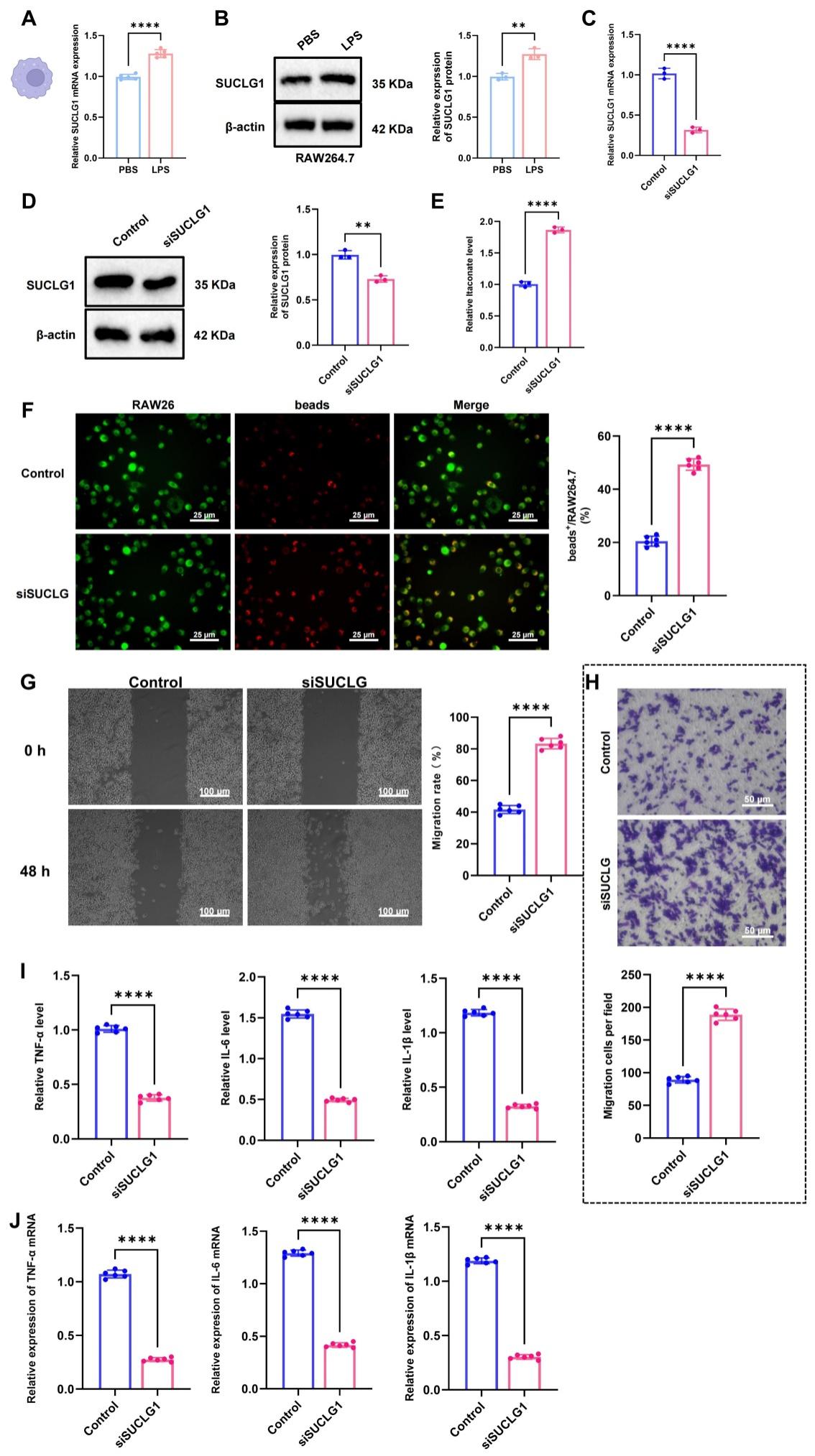
**

**Figure S2. Immune functional reprogramming of macrophages following PEVs@PLGA@si-SUCLG1 treatment.**

**Note:** (A) RT-qPCR analysis of SUCLG1 expression in RAW264.7 macrophages treated with LPS or PBS; (B) Western blot analysis of SUCLG1 protein levels in LPS- or PBS-treated RAW264.7 cells; (C) RT-qPCR analysis of SUCLG1 mRNA expression in different treatment groups; (D) Western blot analysis of SUCLG1 protein expression in various groups; (E) LC-MS quantification of intracellular itaconate accumulation in macrophages; (F) Latex bead phagocytosis assay evaluating macrophage phagocytic capacity (scale bar: 25 µm); (G) Wound-healing assay assessing macrophage migratory ability (scale bar: 100 µm); (H) Transwell assay assessing macrophage chemotaxis (scale bar: 50 µm); (I) ELISA analysis of TNF-α, IL-6, and IL-1β secretion levels; (J) RT-qPCR analysis of TNF-α, IL-6, and IL-1β mRNA levels. Experiments were performed in triplicate; ***p*<0.01, ****p*<0.001, *****p*<0.0001.

**
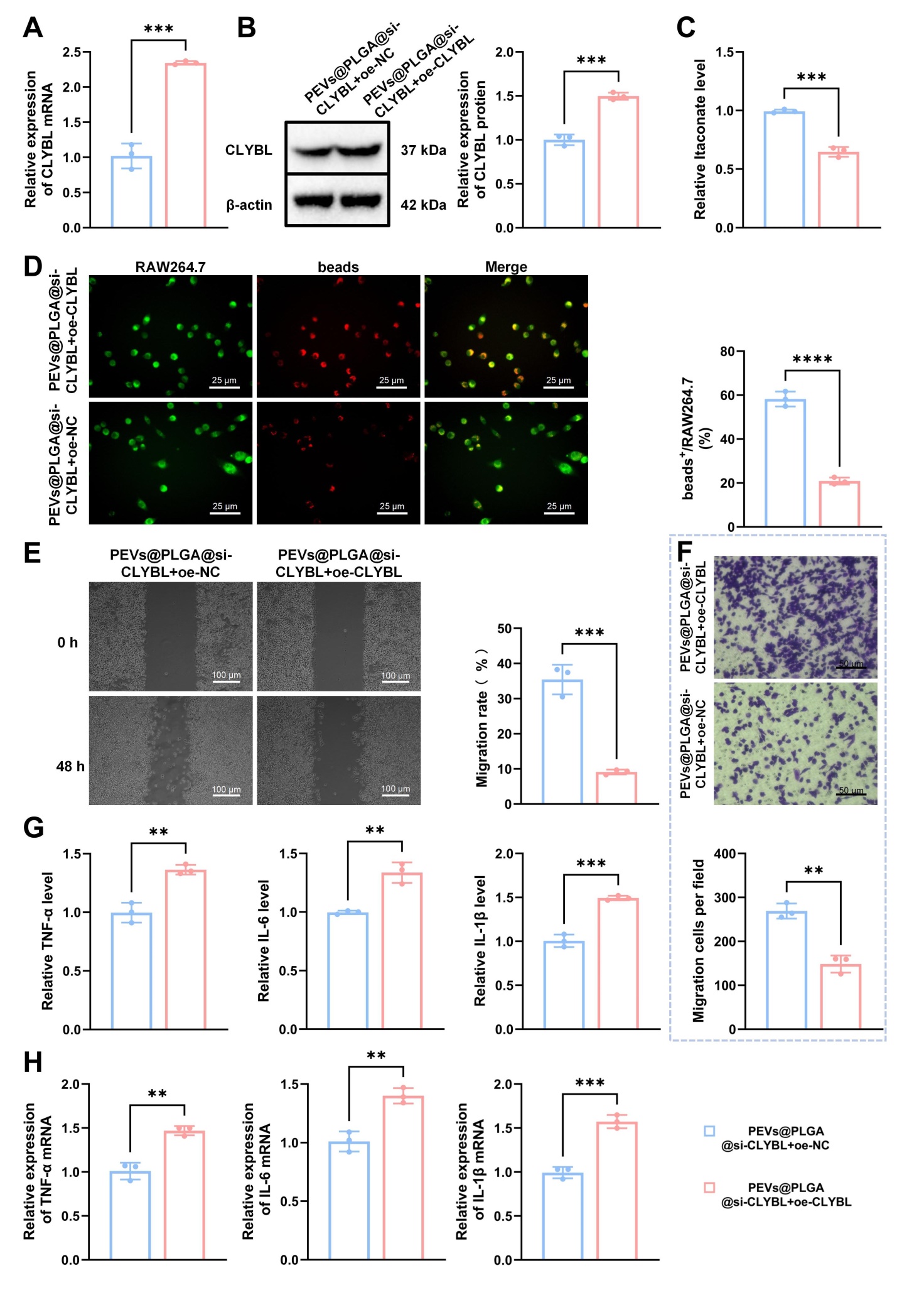
**

**Figure S3. Effects of CLYBL overexpression on macrophage immune function following PEVs@PLGA@si-CLYBL treatment.**

Note: (A) RT-qPCR analysis of CLYBL mRNA levels in macrophages from different groups; (B) Western blot analysis of CLYBL protein expression; (C) LC-MS measurement of intracellular itaconate levels; (D) Latex bead phagocytosis assay to assess macrophage phagocytic capacity (scale bar: 25 μm); (E) Scratch assay to evaluate cell migration (scale bar: 100 μm); (F) Transwell assay to assess chemotactic ability (scale bar: 50 μm); (G) ELISA quantification of TNF-α, IL-6, and IL-1β; (H) RT-qPCR analysis of TNF-α, IL-6, and IL-1β mRNA expression. All experiments were repeated three times. **p*<0.05, ***p*<0.01, ****p*<0.001, *****p*<0.0001.

**
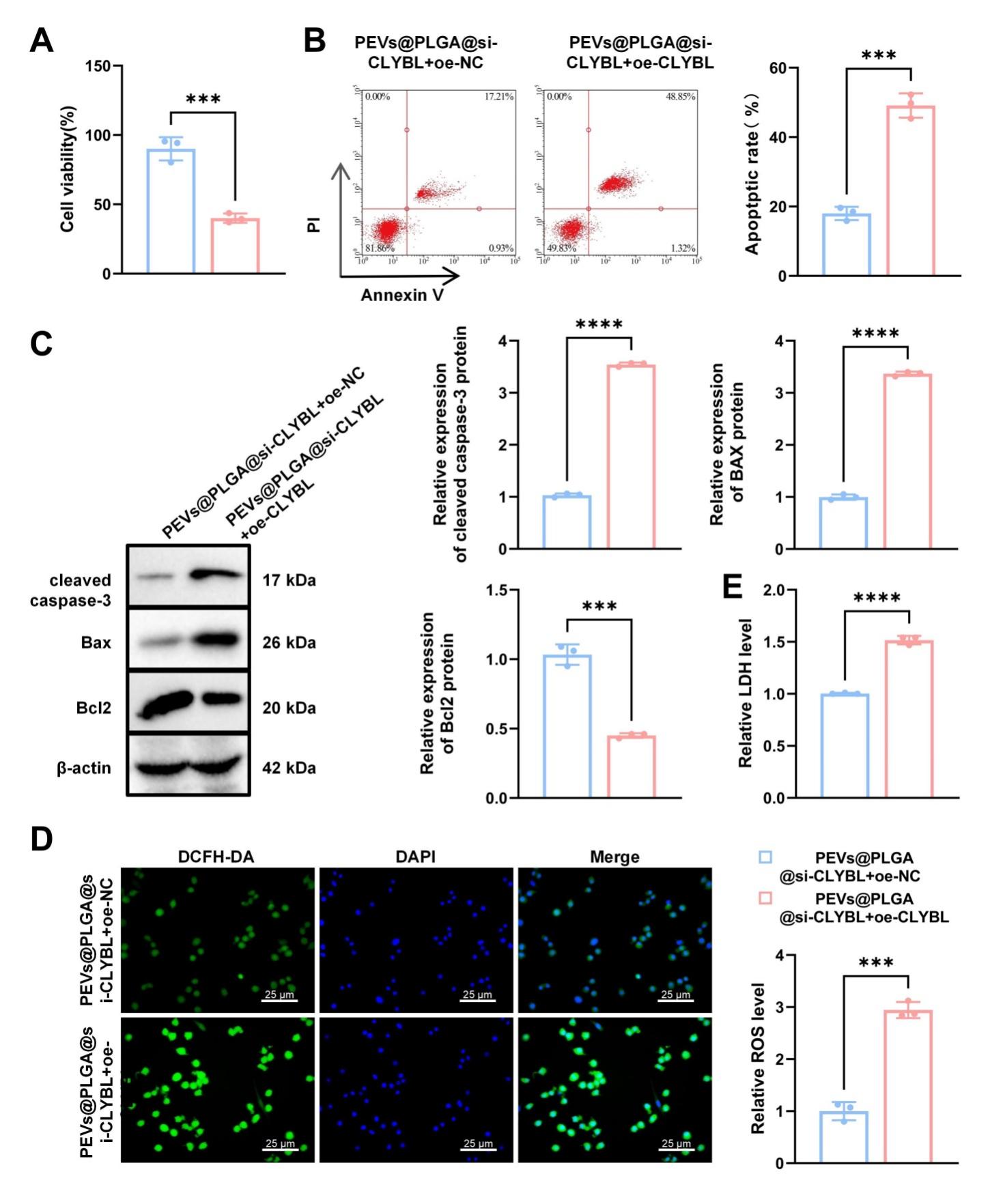
**

**Figure S4. Reversal effects of CLYBL overexpression on PEVs@PLGA@si-CLYBL-treated macrophages and lung epithelial cells.**

Note: (A) CCK-8 assay to compare cell proliferation and viability between the PEVs@PLGA@si-CLYBL group and the PEVs@PLGA@si-CLYBL+oe-CLYBL group; (B) Flow cytometry analysis of apoptosis rates in both groups; (C) Western blot analysis of apoptosis-related protein expression (cleaved caspase-3, Bcl2, Bax) in macrophages; (D) ROS assay to evaluate oxidative stress levels in lung epithelial cells upon CLYBL overexpression (scale bar: 25 μm); (E) LDH release assay to assess membrane damage in response to CLYBL overexpression. All experiments were repeated three times. **p*<0.05, ****p*<0.001, *****p*<0.0001.

**
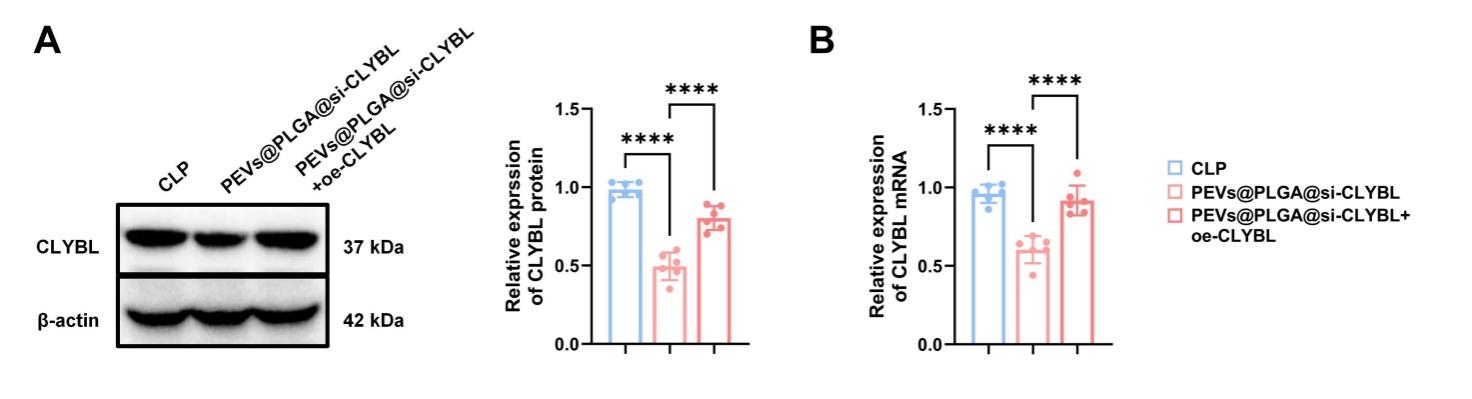
**

**Figure S5. Validation of CLYBL overexpression in lung tissues of septic mice.**

Note: (A-B) Western blot and RT-qPCR analyses of CLYBL expression in lung tissues. All experiments were repeated three times with six animals per group. **p*<0.05, *****p*<0.0001.

**
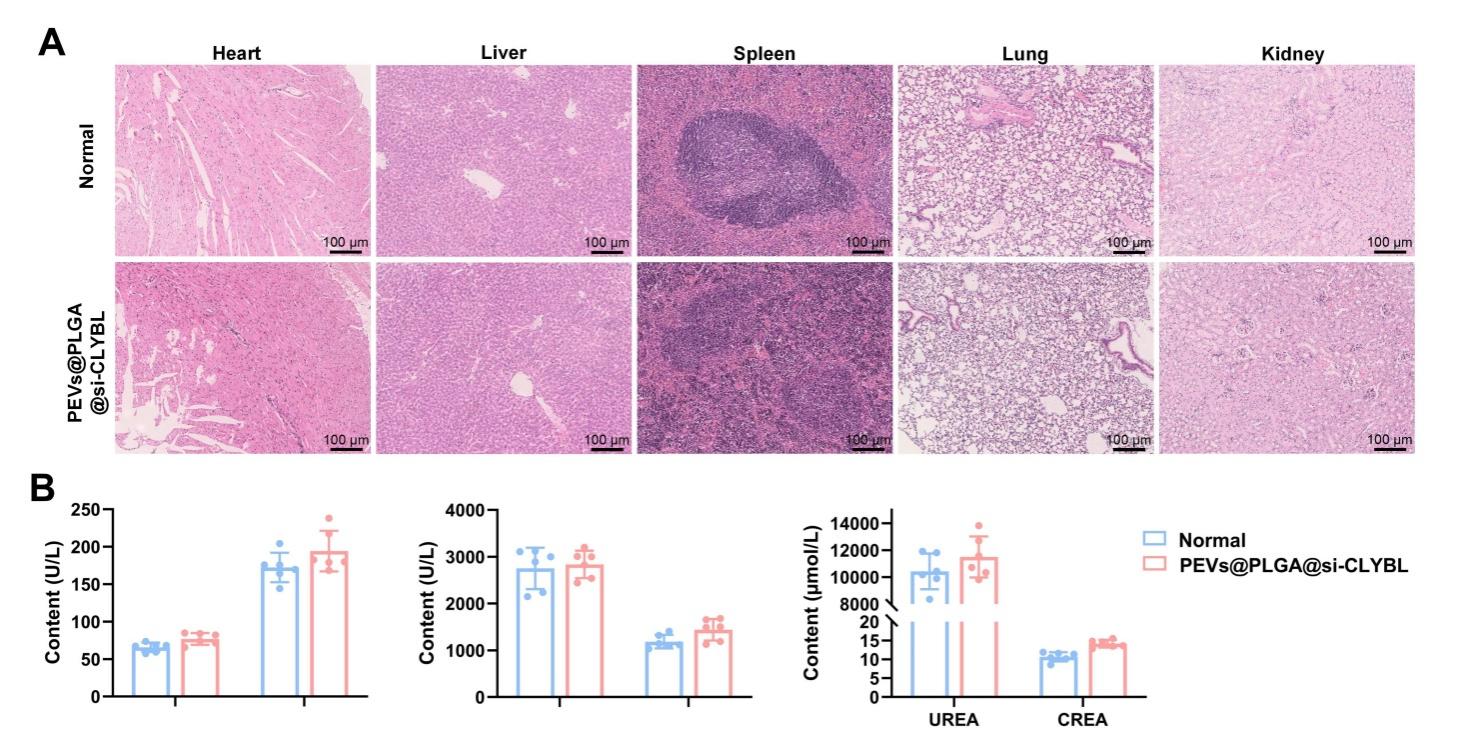
**

**Figure S6. *In vivo* biosafety evaluation of PEVs@PLGA@si-CLYBL nanoparticles.**

Note: (A) H&E staining of major organs (heart, liver, spleen, and kidney) to assess potential pathological damage (scale bar: 100 μm); (B) Automated serum biochemistry analysis of organ function indicators, including CK, LDH, ALT, AST, CREA, and urea. All experiments were repeated three times with six animals per group. **p*<0.05, ***p*<0.01, ****p*<0.001.

**
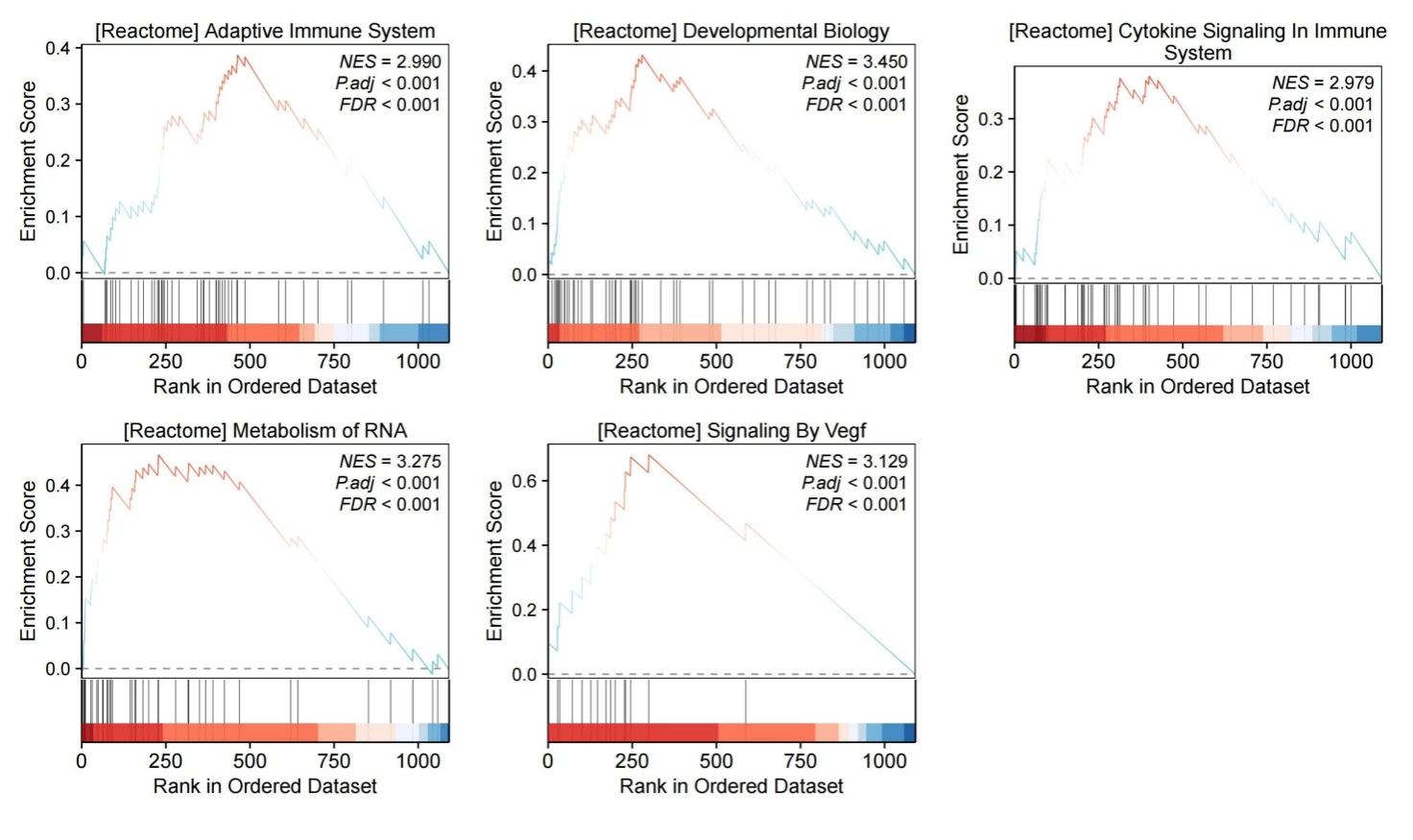
**

**Figure S7. GSEA analysis.**

Note: GSEA visualization of transcriptomic profiles comparing lung tissues from the sepsis group and the treatment group. Sepsis: n=3; Treat: n=3.

**
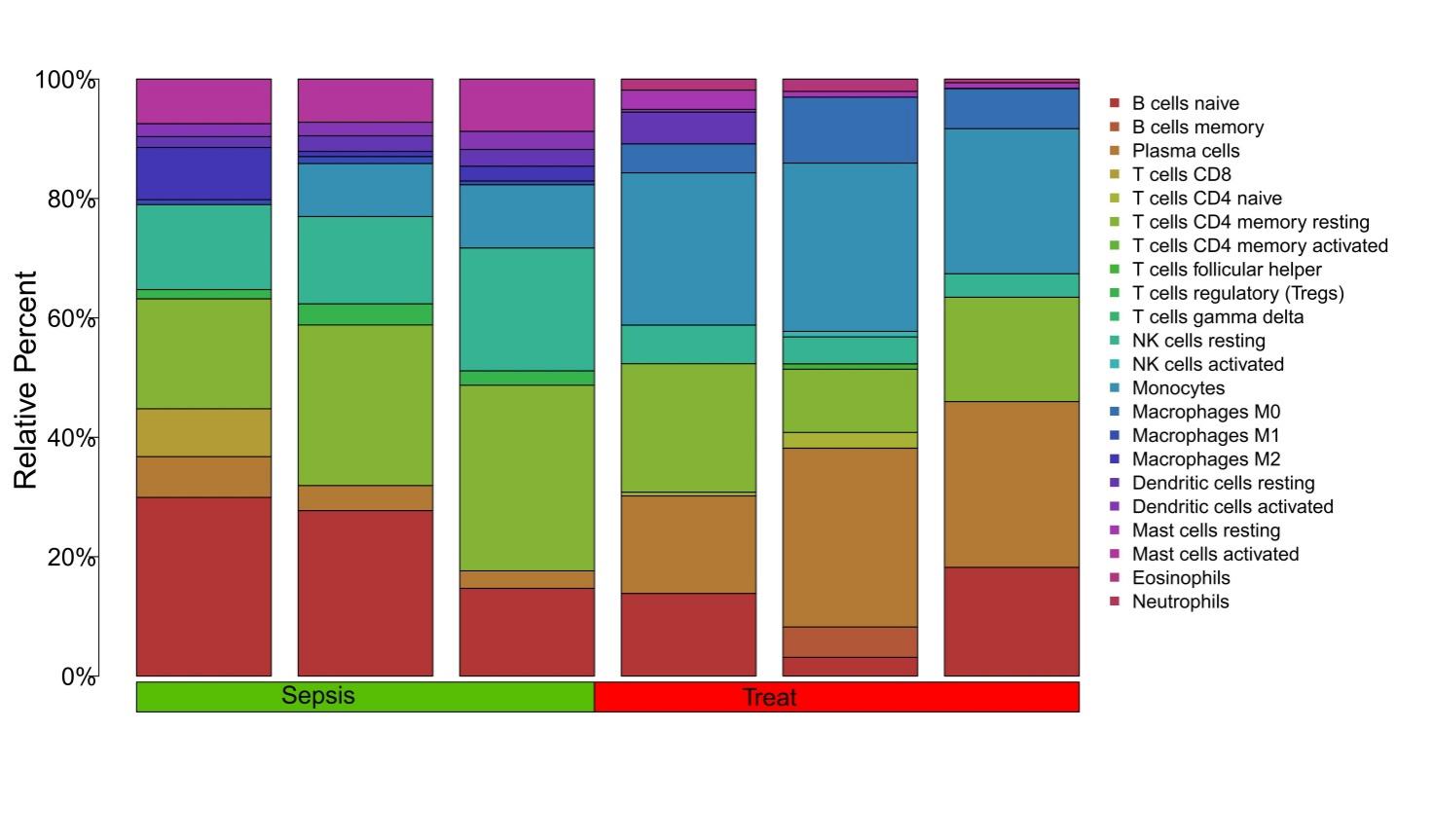
**

**Figure S8. Immune infiltration analysis.**

Note: Composition of 22 immune cell subtypes in lung tissue samples from the sepsis-induced lung injury group and the treatment group. Sepsis: n = 3; Treat: n = 3.
